# Age-dependent impairment of adipose-derived stem cells isolated from horses

**DOI:** 10.1186/s13287-019-1512-6

**Published:** 2020-01-03

**Authors:** Michalina Alicka, Katarzyna Kornicka-Garbowska, Katarzyna Kucharczyk, Martyna Kępska, Michael Rӧcken, Krzysztof Marycz

**Affiliations:** 1 Department of Experimental Biology, Wroclaw University of Environmental and Life Sciences, Norwida 27B, 50-375 Wrocław, Poland; 2 International Institute of Translational Medicine, Jesionowa, 11, Malin, 55-114 Wisznia Mała, Poland; 3 Faculty of Veterinary Medicine, Equine Clinic – Equine Surgery, Justus-Liebig University, 35392 Giessen, Germany

**Keywords:** Aging, Equine adipose-derived mesenchymal stem cells, Endoplasmic reticulum stress, Pro-inflammatory cytokines, Insulin resistance

## Abstract

**Background:**

Progressive loss of cell functionality caused by an age-related impairment in cell metabolism concerns not only mature specialized cells but also its progenitors, which significantly reduces their regenerative potential. Adipose-derived stem cells (ASCs) are most commonly used in veterinary medicine as an alternative treatment option in ligaments and cartilage injuries, especially in case of high-value sport horses. Therefore, the main aim of this study was to identify the molecular alternations in ASCs derived from three age-matched horse groups: young (< 5), middle-aged (5–15), and old (> 15 years old).

**Methods:**

ASCs were isolated from three age-matched horse groups using an enzymatic method. Molecular changes were assessed using qRT-PCR, ELISA and western blot methods, flow cytometry-based system, and confocal and scanning electron microscopy.

**Results:**

Our findings showed that ASCs derived from the middle-aged and old groups exhibited a typical senescence phenotype, such as increased percentage of G1/G0-arrested cells, binucleation, enhanced β-galactosidase activity, and accumulation of γH2AX foci, as well as a reduction in cell proliferation. Moreover, aged ASCs were characterized by increased gene expression of pro-inflammatory cytokines and miRNAs (interleukin 8 (IL-8), IL-1β, tumor necrosis factor α (TNF-α), miR-203b-5p, and miR-16-5p), as well as apoptosis markers (p21, p53, caspase-3, caspase-9). In addition, our study revealed that the protein level of mitofusin 1 (MFN1) markedly decreased with increasing age. Aged ASCs also displayed a reduction in mRNA levels of genes involved in stem cell homeostasis and homing, like TET-3, TET-3 (TET family), and C-X-C chemokine receptor type 4 (CXCR4), as well as protein expression of DNA methyltransferase (DNMT1) and octamer transcription factor 3/4 (Oct 3/4). Furthermore, we observed a higher splicing ratio of XBP1 (X-box binding protein 1) mRNA, indicating elevated inositol*-*requiring enzyme 1 (IRE-1) activity and, consequently, increased endoplasmic reticulum (ER) stress. We also observed reduced levels of glucose transporter 4 (GLUT-4) and insulin receptor (INSR) which indicated impaired insulin sensitivity.

**Conclusions:**

Obtained data suggest that ASCs derived from horses older than 5 years old exhibited several molecular alternations which markedly limit their regenerative capacity. The results provide valuable information that allows for a better understanding of the molecular events occurring in ASCs in the course of aging and may help to identify new potential drug targets to restore their regenerative potential.

**Graphical abstract:**

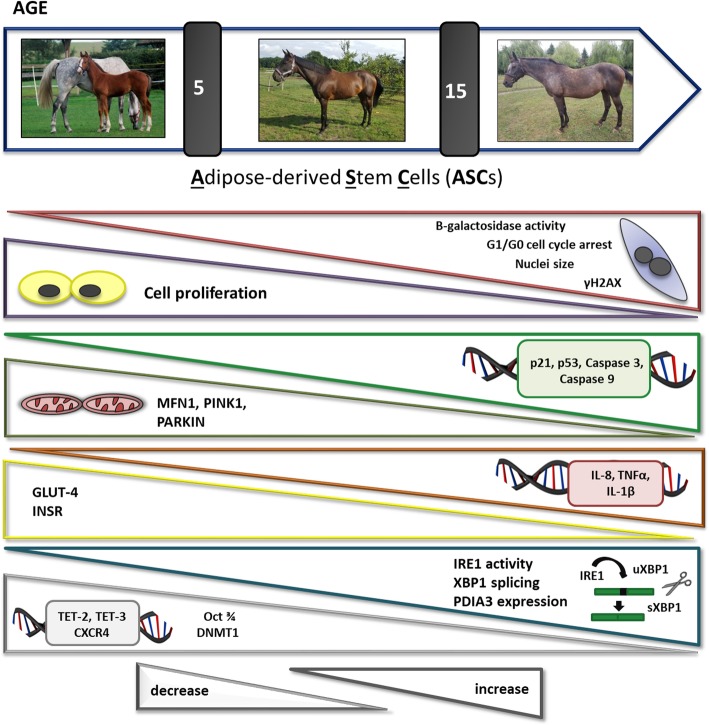

## Introduction

Mesenchymal stem cells (MSCs) are adult stem cells with the ability to differentiate into cells of mesodermal lineage such as adipocytes, osteocytes, and chondrocytes. MSCs were originally described in the bone marrow by Friedenstein and coworkers in the 1960s and 1970s, who identified MSCs as clonal osteogenic progenitors and showed their colony-forming capacity [1]. Currently, MSC can be isolated from several vascularized types of tissue like dental pulp (DPSCs), adipose tissue (ASC), and Wharton’s jelly (WJ-MSCs) [2]. MSC exhibits expression of specific surface antigen, such as CD105, CD73, and CD90, and lacks expression of hematopoietic and endothelial markers (CD45, CD34, CD11, CD14) [3]. Additionally, they express pluripotency markers like Oct-4 and SOX-2 [4]. One of the main points of interest regarding MSC in cellular therapy is graft and host compatibility. Extensive evidence has shown that MSC exhibits low expression of MHC class I and lack of MHC II along with the secretion of soluble immunomodulatory agents that regulate B cell and T cell function, which makes them a promising tool in regenerative medicine [5, 6]. Interestingly, several studies have reported that only a small fraction of transplanted MSCs engraft in the host body. The fact suggests that paracrine soluble factors produced and secreted by MSCs have a specific responsibility for tissue repairing [7, 8].

Aging is an inevitable physiological consequence of living organisms. Mammalian aging is characterized by the complex cellular processes called cellular senescence. Cellular senescence does not only affect mature specialized cells but also stem cells. As mentioned previously, adult stem cells, such as MSC, are found throughout the body. Their primary roles are to maintain and repair the damaged tissue where they are found; however, their regenerative potential can be diminished by several factors, such as age [9, 10] and metabolic disorders [11–14]. Senescence causes lower cell proliferation and viability, dramatic mitochondria deterioration, and insufficient protection against oxidative stress. Moreover, senescent cells secrete pro-inflammatory and matrix-degrading factors called senescence-associated secretory phenotype (SASP) [15]. Recent experimental evidence has shown an increased inflammatory potential of adipose tissue in aged animals by overproduction of interleukin 6 (IL-6), IL-8, IL-1β, and tumor necrosis factor α (TNF-α) [16–18] causing insulin resistance [18, 19]. It has been shown that an age-related increase in visceral adipose tissue leads to increased pro-inflammatory cytokine levels that interfere with the insulin cascade pathway over the course of aging [20]. Chronic inflammation enhances oxidative stress by overproduction of reactive oxygen and nitrogen species (RONS) and decreases cellular antioxidant capacity [21]. Imbalance between the production of RONS and antioxidant enzyme activities, such as superoxide dismutase (SOD) and catalase (CAT), leads to oxidative damage to DNA, proteins, and lipids [22]. In eukaryotic cells, mitochondria play a main role in RONS generation. What is more, excessive oxidative metabolism causes mitochondrial DNA (mtDNA) damage, resulting in mitochondrial deterioration. It has been observed that excessive production of RONS is accompanied by rapid mitochondrial fragmentation, which indicates a high correlation between mitochondria fission and mitochondrial-mediated oxidative stress [23, 24]. Several lines of evidence support a strong link between cellular senescence and endoplasmic reticulum (ER) stress. ER is a membranous tubular network that plays a crucial role in calcium homeostasis and protein and lipid biosynthesis in eukaryotic cells. Under normal conditions, the newly synthesized proteins enter the ER lumen and then undergo folding and post-translational modification. Maintaining ER homeostasis is critical for cell metabolism and survival. Many disturbances, such as changes in redox balance, hypoxia, alternations in calcium homeostasis, nutrient (mainly glucose) deprivation, and failure of folding and post-translational modification, as well as increase in general protein synthesis (necessary for the production of the SASP) can induce unfolded protein response (UPR) [25, 26]. The UPR consists of three parallel arms sensed by ER-resident transmembrane proteins, which are referred to as protein kinase RNA-like endoplasmic reticulum kinase (PERK), activating transcription factor 6 (ATF6), and inositol*-*requiring enzyme 1 (IRE-1α). PERK and IRE1-1α are activated after self-transphosphorylation, whereas ATF6 is activated through proteolytic cleavage. The induction of the PERK-governed signaling pathway is almost simultaneous with the ER stress enablement, followed by the induction of ATF6 and then IRE1 signaling branches [11, 25]. Activated IRE1 catalyzes the alternative splicing of X-box binding protein 1 (XBP1). Spliced XBP1 (sXBP1) undergoes translation and forms an active transcription factor involved in the regulation of ER protein folding, lipid biogenesis, and ER-associated degradation (ERAD). UPR is considered to be a pro-survival mechanism, but its prolonged activation may promote apoptosis [27]. Cell proliferation leads to progressive telomere erosion that may trigger a tumor suppressor protein 53 (p53) and cause upregulation of the p53 transcriptional target 21 (p21). This mechanism prevents entry into the synthesis phase (S phase) of the cell cycle [28]. In vitro senescent cells are characterized by increased cell and nuclei size, as well as enhanced enzymatic activity of the lysosomal hydrolase senescence-associated β-galactosidase (SA-β-gal) [28, 29].

With regard to their biological importance and clinical applications, there are almost 1000 clinical trials using MSCs in several diseases including orthopedic, autoimmune, and cardiovascular disorders [30]. Interestingly, MSCs have been used as a therapeutic agent in horses for over 15 years [31]. In comparison with autologous MSC transplantation, allogeneic MSCs can cause both cellular and humoral alloimmunity in humans and horses by induction of specific alloantibodies which increase the risk of possible rejection [32, 33]. Thus, it is crucial to determine whether donor age does not affect the therapeutic potential of these cells. The main purpose of this study was to investigate the proliferation capacity, cell morphology, and viability of ASCs derived from three age donor groups: (1) < 5-, (2) 5–15-, and (3) > 15-year-old horses. The special attention has been paid toward oxidative and ER stress, mRNA and miRNA expression profile, secretory activity, protection against oxidative damage, and mitochondrial dynamics deterioration.

## Materials and methods

All of the reagents used in the study were purchased from Sigma-Aldrich (CA, USA), unless indicated otherwise.

### Cell isolation and culture

Equine subcutaneous adipose tissue was collected from both male and female individuals (with female predominance) from the tail base of horses, with the approval of the Local Ethical Committee in Wroclaw (84/2018). Horses (age-matched (1–23 years; mean ± SD, 9.6 ± 6.1 years)) were divided into three age groups: young (< 5; age range 1–4, *n* = 6, mean age 3 ± 0.9 years), middle-aged (5–15, age range 5–15, *n* = 6, mean age 9.7 ± 2.5 years), and old (> 15; age range 15–23, *n* = 6, mean age 18.3 ± 1.5 years). Experimental horses were warmblood horses, coming from various horse breeding and used in small sports training in jumping direction. Horses were fed with commercial feeds, which doses were selected individually and depend on actual energy demand. The hay was given without restrictions. Horses had regular veterinarian controls and did not suffer from equine metabolic syndrome (EMS). The intensity of training depends on age and condition.

Tissue fragments were washed with phosphate-buffered saline (PBS) solution supplemented with 1% of antibiotic mix (penicillin/streptomycin (PS)) and chopped into smaller pieces before enzymatic digestion. Then, tissue pieces were incubated in collagenase type I solution (1 mg/ml) for 40 min at 37 °C. Digested samples were centrifuged at 1200×*g* for 10 min at RT. Obtained cell pellets were resuspended in Dulbecco’s modified Eagle’s medium (DMEM) low glucose supplemented with 10% of fetal bovine serum (FBS) and 1% PS solution and transferred to the T25 culture flask (Nunc, USA). The medium was refreshed every 2–3 days. The cells were passaged when grown to 80% confluence using recombinant cell-dissociation enzyme TrypLE Express (Life Technologies, USA). At passage 3, ASC phenotype was confirmed by analysis of the expression of CD44, CD90, and CD45, and their tri-lineage differentiation potential was assessed, as previously shown [34].

### Assessment of cell proliferation

Cell proliferation rate was estimated using TOX-8 resazurin-based in vitro toxicology assay kit after 24, 48, 96, and 144 h of culture. For the assay, culture media were replaced with fresh media supplemented with 10% v/v resazurin dye, and incubation was carried out for 2 h at 37 °C in the CO_2_ cell culture incubator (Thermo Fisher, USA). The supernatants were subsequently transferred to 96-well plate (Greiner Bio-One, Austria) in 100 μl per well and measured using spectrophotometer (Epoch, Biotek, Germany) at a wavelength of 600 nm and 690 nm reference length. Population doubling time (PDT) was determined using an online algorithm software [35].

### ASC morphology and ultrastructure

Cell morphology was evaluated using scanning electron microscopy (SEM) and fluorescent microscopy. In the SEM analysis, cells were fixed with 4% paraformaldehyde (PFA) for 45 min at RT, rinsed with distilled water, and dehydrated in graded ethanol series (ethanol concentration from 50 to 100%, every 5 min). Then, the samples were sprinkled with gold (ScanCoat 6, UK) and observed using SE1 detector at 1 kV of filament tension.

Mitochondria visualization was performed using MitoRed dye in live cells. First, the supernatant was replaced with fresh culture media containing 0.1% of MitoRed, and cells were incubated for 30 min at 37 °C. Then, cells were fixed with 4% PFA as described above, washed with PBS, and counterstained with 4′,6-diamidino-2-phenylindole (DAPI) to visualize the cell nuclei.

F-actin was visualized in fixed and permeabilized cells using Phalloidin Atto 590. Cells were fixed with 4% PFA, washed and permeabilized with 0.2% Tween 20 in PBS for 15 min, and incubated with Phalloidin Atto 590 solution in PBS (1:1000) for 45 min at RT in the dark. The cell nuclei were counterstained using DAPI.

Proliferation was evaluated using Ki-67 nuclear antigen staining. ASCs were rinsed with PBS, fixed with 4% PFA permeabilized with 0.2% Tween 20 in PBS for 15 min, washed again, and blocked using a solution of 1% BSA and 22.52 mg/ml glycine in PBST for 20 min to avoid unspecific binding of the antibody. Then, samples were incubated with primary anti-Ki-67 antibody (dilution 1:100 in 1% BSA in PBST solution) (Abcam, UK) overnight at 4 °C, rinsed three times with PBS, and incubated with secondary Atto 590-conjugated secondary anti-rabbit antibody (1:1000) (Abcam, UK) for 1 h at RT in the dark. Before DAPI staining, the samples were washed three times with PBS.

The endoplasmic reticulum structure was visualized using the anti-PDIA3 (protein disulfide-isomerase A3) antibody (Novus Biologicals, UK). Cells were fixed with 4% PFA, rinsed with PBS and permeabilized with 0.2% Tween 20 in PBS for 15 min, washed again, and blocked with 10% goat serum for 30 min. Then, cells were incubated with an anti-PDIA3 antibody (1:100 dilution in PBS) overnight at 4 °C. Atto 590-conjugated anti-rabbit secondary antibodies were used to detect the signal.

Glucose transporter (GLUT-4) staining was carried out as described above. Cells were fixed with 4% PFA and washed with PBS. After the incubation, cells were washed again and then blocked with 10% goat serum for 30 min followed by incubation with anti-GLUT-4 primary antibodies (dilution 1:100) overnight at 4 °C. We used Atto 590-conjugated anti-rabbit secondary antibodies to detect the signal. The incubation was carried out for 1 h at RT. The nuclei were counterstained with DAPI. Cells were observed and imaged using a confocal microscope (Observer Z1 Confocal Spinning Disc V.2 Zeiss with live imaging chamber). Pictures were analyzed using ImageJ software.

### Analysis of mRNA and miRNA expression profiles

In order to assess the mRNA and miRNA expression, total RNA was extracted from cells using phenol-chloroform method, as previously described by Chomczynski and Sacchi [36]. RNA quality and quantity were evaluated spectrophotometrically (Epoch, Biotek, Germany). For mRNA expression analysis, 150 ng of total RNA was used for cDNA synthesis with random hexamers using RevertAid First Strand cDNA Synthesis Kit (Thermo Fisher Scientific, USA) followed by gDNA digestion with DNase I RNase-free Kit (Thermo Fisher Scientific, USA). Both reactions were performed using T100 Thermal Cycler (Bio-Rad, USA). Each qPCR reaction mixture contained 500 nM of each specific primer (see Additional file 1), 7.5 μl of SensiFast SYBR & Fluorescein Kit (Bioline, UK), and 1 μl of cDNA in a total volume of 10 μl. All qPCR reactions were conducted using CFX Connect™ Real-Time PCR Detection System (Bio-Rad, USA). Expression data were normalized to the geometric mean of GAPDH used as a housekeeping gene to control the variability in the expression levels and were analyzed using the 2^−ΔΔCT^ method described by Livak and Schmittgen [37]. Furthermore, the ratio of BCl-2/BAX expression was determined by dividing the ΔΔCT of genes.

The splicing of XBP1 was detected by standard quality RT-PCR using primers designed previously by Cassimeris et al. [38]. The PCR products were run in 2% gel and separated to visualize a 26-bp shift. Similarly to other genes, GAPDH was used as the normalization control.

To determine the miRNA expression, 500 ng of total RNA was served for gDNA digestion as described above. Next, RNA was polyadenylated and cDNA was synthesized using the Mir-X miRNA First-Strand Synthesis Kit (Clontech Laboratories, Inc., USA) according to the manufacturer’s protocol. Each qPCR reaction was performed in a total reaction mixture volume of 25 μl. The reaction included the initial denaturation at 95 °C for 10 s, followed by 55 cycles of 95 °C for 5 s and annealing temperature of 60 °C for 20 s with a single fluorescence measurement. Expression data were normalized using the 2^−ΔΔCT^ method in relation to the U6 snRNA used as a housekeeping gene (Table 1).

**Table 1:** List of miRNA with accession numbers and sequence of primers in qPCR

miRNAs	Primers sequences 5′→3′	Accession no.
miR-17-5p	CAAAGTGCTTACAGTGCAGGTAG	MIMAT0000070
miR-24-3p	TGGCTCAGTTCAGCAGGAACAG	MIMAT0000080
miR-146a-5p	TGAGAACTGAATTCCATGGGTT	MIMAT0000449
miR-101-1/2	TACAGTACTGTGATAACTGAA	MI0000103
miR-21	TAGCTTATCAGACTGATGTTGA	MIMAT0000530
miR-203b-3p	TTGAACTGTTAAGAACCACTGGA	MIMAT0019814
miR-16-5p	TAGCAGCACGTAAATATTGGCG	MIMAT0000069

### Western blotting

Cells were detached from culture dishes and homogenized in RIPA buffer with protease inhibitor cocktail. Cell lysates were centrifuged for 20 min at 14000×*g* (4 °C). Supernatants were collected in fresh tubes and stored at − 80 °C. The protein concentration was determined using the Pierce™ BCA Protein Assay Kit (Life Technologies, USA). Cell lysates were mixed with 4× Laemmli loading buffer (Bio-Rad, USA) and incubated for 5 min at 95 °C. Samples were subjected to SDS-polyacrylamide gel electrophoresis (15 μg of proteins per well) at 100 V for 90 min in Tris/glycine/SDS buffer using Mini-PROTEAN Tetra Vertical Electrophoresis Cell (Bio-Rad, USA) and transferred onto polyvinylidene difluoride (PVDF) membranes (Bio-Rad, USA) using a transfer apparatus Mini Trans-Blot® Cell (Bio-Rad, USA) at 100 V, 250 mA for 1 h at 4 °C in Tris/glycine buffer/methanol. The membranes of non-phosphorylated proteins were blocked using 5% non-fat milk in TBST, whereas in order to detect p-γH2AX protein membranes were blocked in 5% bovine serum albumin (BSA) in TBST. Each protein was detected by overnight incubation at 4 °C with primary antibodies (Table 2) and HRP-conjugated secondary antibodies (dilution 1:2500 in TBST, 1 h incubation). Chemiluminescent signals were detected using ChemiDoc MP Imaging System (Bio-Rad, USA) and quantified with Image Lab Software (Bio-Rad, USA).

**Table 2:** List of proteins with manufacturers, catalog numbers, and dilution used in western blotting

Protein	Dilution	Manufacturer, catalog no.
p-γH2AX	1:1000	Abcam, ab11174
DNMT1	1:1000	Sigma Aldrich, D4567
MFN1	1:1000	Biorbyt, orb11040
MFF	1:1000	Biorbyt, orb325479
GLUT-4	1:1000	Sigma Aldrich, G4173
INSR	1:1000	Biorbyt, orb379724
Oct 3/4	1:1000	Sigma Aldrich, O8389
β-Actin	1:5000	Sigma Aldrich, A5441

*P-γH2AX* phospho-H2AX, *DNMT1* DNA methyltransferase 1, *MFN1* mitofusin 1, *MFF* mitochondrial fission factor, *GLUT-4* glucose transporter 4, *INSR* insulin receptor

### Evaluation of β-galactosidase activation

Detection of cellular senescence the lysosomal enzyme senescence-associated β-galactosidase (SA-β-gal) activity was performed using Senescence Cells Histochemical Staining Kit in accordance with the manufacturer’s instruction. Briefly, cells were fixed with formaldehyde-based fixation buffer (RT, 6 min), washed with PBS, and incubated with X-gal solution containing 5-bromo-4-chloro-3-indolyl-b-d-galactopyranoside overnight at 37 °C. Blue-stained senescent cells were observed and imaged using an invert microscope (Leica, Germany). At least 100 cells were counted to determine the percentage of SA-β-gal-positive cells.

### Analysis of ROS accumulation using flow cytometry-based system

Intracellular ROS were detected using a Muse® Oxidative Stress Kit based on dihydroethidium (DHE). The assay allowed to distinguish two populations of cells: ROS(−) live cells and ROS(+) cells exhibiting a high level of intracellular ROS. The procedure was performed in accordance with the protocol provided by the supplier. Relative percentage of ROS(−) and ROS(+) cells were obtained using Muse™ Cell Analyzer (Merck, Germany).

### Determination of catalase activity

The CAT activity ASCs were measured using Catalase (CAT) Assay Kit (MyBioSource, CA, USA). The procedure was performed according to the manufacturer’s protocol.

### Cell cycle analysis

Cell cycle analysis was performed with Muse™ Cell Cycle kit (Milipore Corp., MA, USA) according to the manufacturer’s protocol. The cell cycle distribution was determined using the flow cytometry-based system Muse™ Cell Analyzer (Merck, Germany).

### Estimation of p53 protein levels using ELISA method

Intracellular concentrations of p53 were determined in lysates from cell culture using ELISA kits purchased from MyBioSource (CA, USA). The analysis was performed in accordance with the manufacturer’s instructions.

### Statistical analysis

The results are presented as means ± SD. Statistical comparison between the groups was conducted using the one-way ANOVA (and non-parametric) test, followed by Tukey’s test for post hoc comparison. Each qRT-PCR and western blot result was normalized to ASC_< 5_ as control. Differences were considered statistically significant at *p < 0.05, **p < 0.01, and ***p < 0.001**.**

## Results

### Proliferative potential of equine ASCs declines with increasing age

**Fig. 1:**
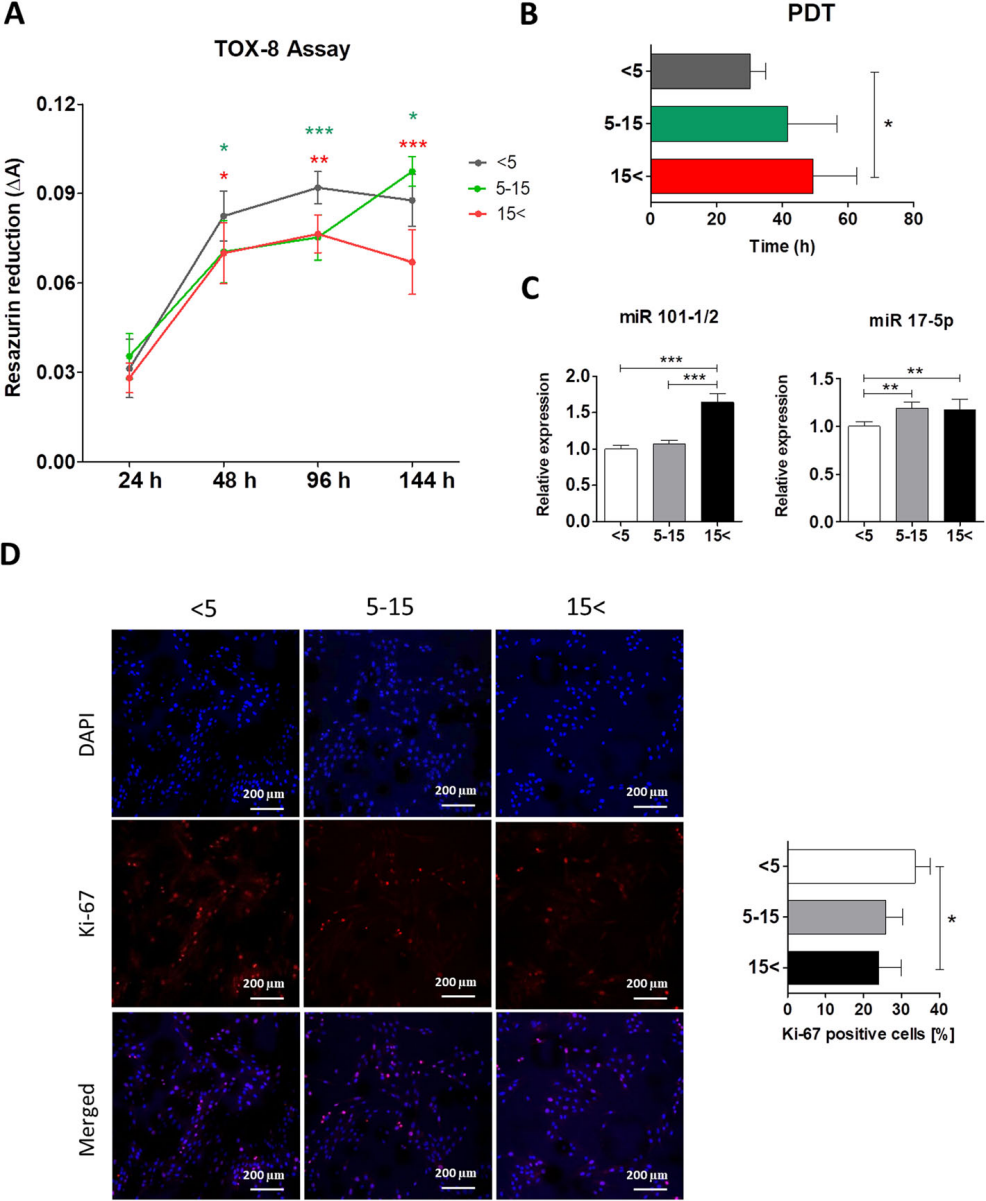
Analysis of proliferation of ASCs for the three age donor groups (0–5 years, 5–15 years, > 15 years). **a** Cell proliferation was assessed, and **b** PDT was quantified using resazurin-based TOX-8 assay. The red asterisk refers to the comparison of the < 5 and 5–15 groups to > 15, whereas the green asterisk refers to the comparison of the < 5 and < 15 groups to 5–15. **c** Relative expression of proliferation-associated miRNAs was detected by the qRT-PCR method. **d** Representative images of immunofluorescence staining for Ki-67 (red) and nuclei (blue). Ki-67 expression in cells was presented as Ki-67-positive cell percentage. A positive colocalization of Ki-67 in the nucleus is indicated by pink signals (merge) due to overlap of Atto 590 (Ki-67) and DAPI staining. Results expressed as mean ± SD. Statistical significance is indicated with asterisks: *p < 0.05, **p < 0.01, ***p < 0.001 using one-way ANOVA (and non-parametric) test

High proliferation capacity is a fundamental characteristic of MSC crucial for expansion and self-renewal and defines stem cell degree of stemness. Cell growth can be measured using PDT value, which is recommended by Cell Products Working Party (European Medicine Agency (EMA)) to determine the time for cell in culture [39]. The proliferative potential of ASCs was characterized by resazurin-based TOX-8 assay over the 6-day culture period. We observed a significant increase in the proliferation of ASCs isolated from young individuals (ASC_< 5_) after 24, 48, 96, and 144 h of culture (Fig. 1a). The high proliferation potential of ASC_< 5_ was confirmed by low PDT value. ASC_< 5_ had significantly shorter PDT when compared with ASC_15< _ (*p* < 0.05) (Fig. 1b). Moreover, we examined the relative expression of proliferation-related miRNA in ASCs using qRT-PCR. The results indicated that increasing age was positively correlated with the expression of both miR-101-1/2 and miR-17-5p. The expression of miR-101-1/2 was significantly higher in ASC_15< _ compared with ASC_< 5_ and ASC_5–15_ (*p* < 0.01). In addition, we detected overexpression of miR-17-5p in ASC_5–15_ and ASC_15< _ (*p* < 0.001) (Fig. 1c). Furthermore, we performed immunofluorescence staining in order to determine the expression of Ki-67, a widely known proliferation marker. Ki-67 is detectable during all active cell cycle phases G1, S, G2, and M but is not expressed in resting cells (G0). Our study revealed a significantly higher expression of Ki-67 cells in young ASCs compared with old individuals (*p* < 0.05) (Fig. 1d). The obtained results suggest that ASC from young horses (< 5 years old) exhibit a more rapid proliferation rate compared with ASCs from older animals. Interestingly, we did not observe significant differences in proliferation between the ASC_5–15_ and ASC_15< _ groups.

### Cellular senescence increasing with horse donor age

**Fig. 2:**
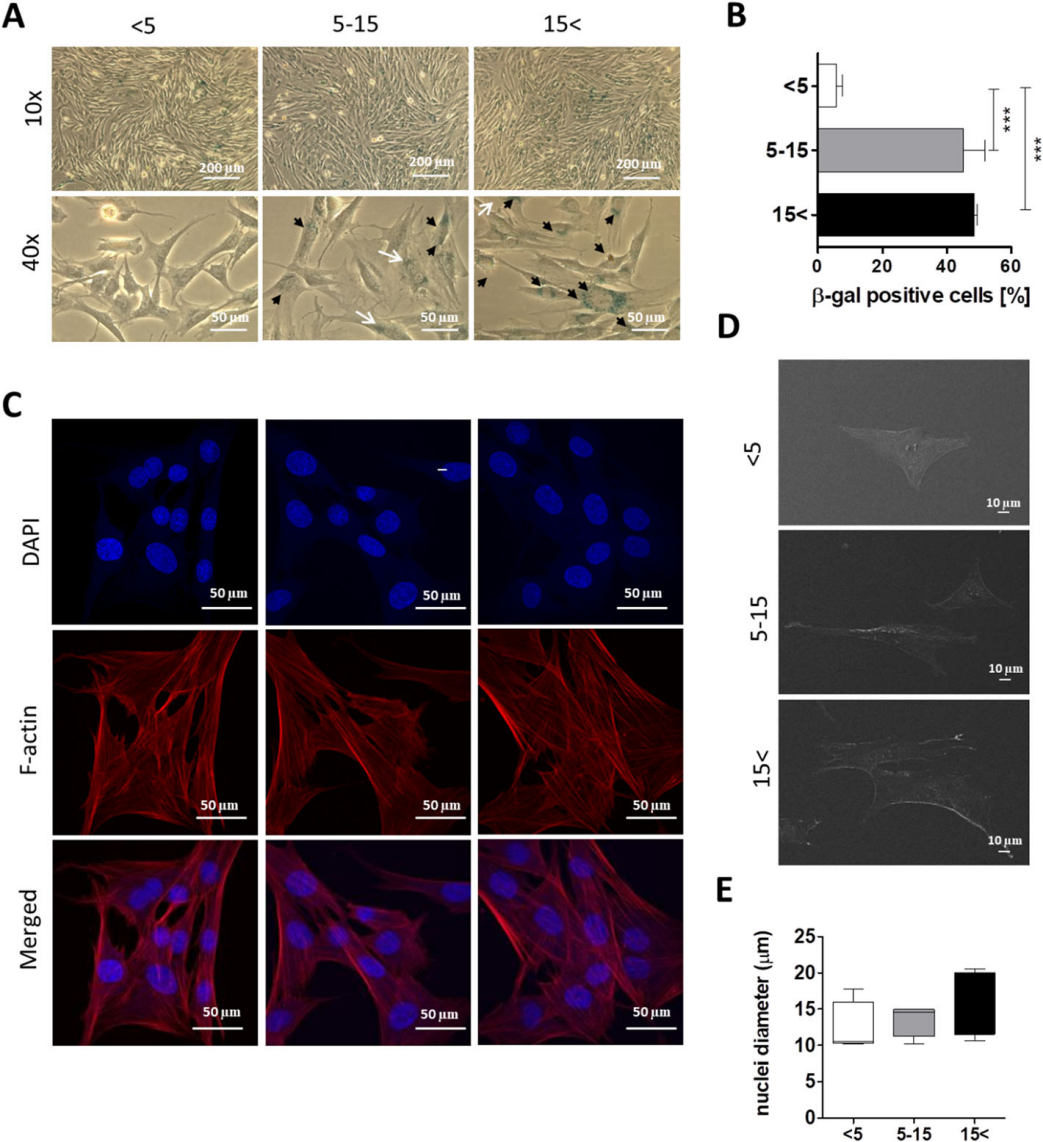
Morphological alternations caused by aging in ASCs. **a** Representative images of senescence-associated SA-β-gal assay. Black arrows indicate SA-β-gal-positive cells (blue), whereas white arrows show binucleated cells. **b** Quantification of SA-β-gal-positive cells (blue). **c** Representative images of Phalloidin Atto 590 staining for F-actin. **d** SEM images of ASCs isolated from the three age groups (ASC_< 5_, ASC_5–15_, ASC_15< _). **e** Mean diameter of the cell nuclei measured on the basis of SEM images. Results expressed as mean ± SD. Statistical significance is indicated with asterisks: *p < 0.05, **p < 0.01, ***p < 0.001 using one-way ANOVA (and non-parametric) test

In light of the connection between cellular senescence and horse donor age, we decided to evaluate the activity of senescence-associated β-galactosidase (SA-β-gal), a well-known and widely accepted marker of the cellular senescent phenotype in vitro. Analysis of SA-β-gal staining showed an increase in the number of β-gal-positive cells in both ASC_5–15_ and ASC_15< _ (black arrows) (*p* < 0.001) (Fig. 2a, b). Furthermore, lots of β-gal-positive cells exhibited binucleated morphology (white arrows) (Fig. 2a). What is more, SEM images showed that ASC_15< _ were characterized by much “flattened” morphology and the nuclei positioned at the cell periphery (Fig. 2d). Although we did not observe significant differences in nuclei size between the experimental groups (Fig. 2e), there is a growing body of evidence that the cell enlargement may be caused by an increase in nuclear diameter [40]. Phalloidin staining showed that F-actin filaments are widely distributed through the cells of each experimental group (Fig. 2c). The results indicate that horse donor age significantly affects ASC senescence. Interestingly, there were no significant differences in β-gal activity between the middle-aged and old groups.

### Age-related changes in DNA content and its structure

**Fig. 3:**
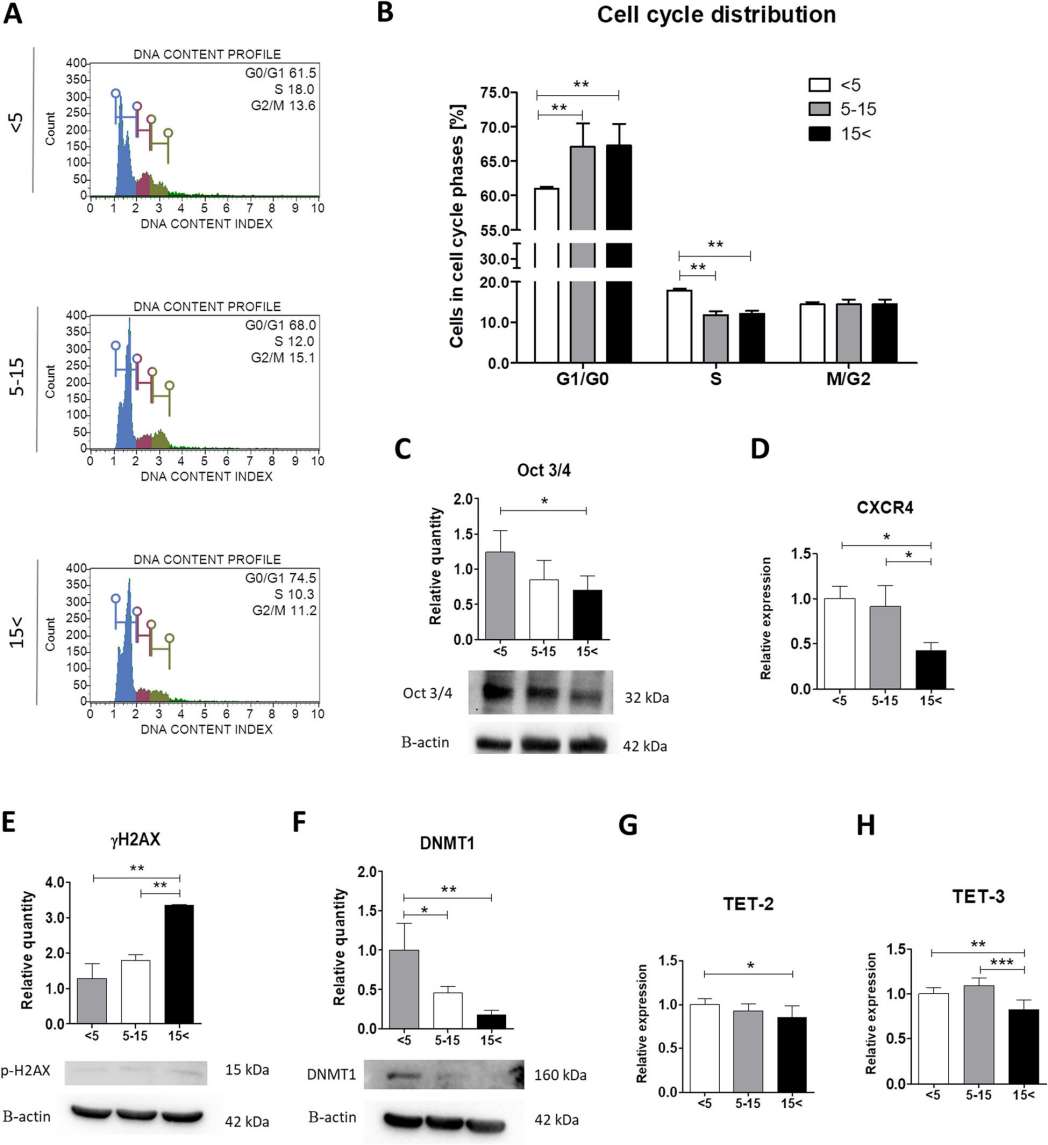
Age-associated changes in the cell cycle distribution (**a**, **b**), protein level of Oct 3/4 (**c**), mRNA level of CXCR4 (**d**), accumulation of γH2AX (**c**), expression of DNMT1 (**f**), and transcript levels of TET-2 and TET-3 (**g**, **h**). Cell cycle distribution was determined using a flow cytometry-based system Muse™ Cell Analyzer. The levels of Oct 3/4, γH2AX, and DNMT1 were estimated with the western blot method. Relative quantity of proteins was estimated using Image Lab software after normalization with β-actin (loading control). Representative blots are shown on the bottom panel. Alternations in the mRNA expressions of CXCR4 (**c**), TET-2 (**g**), and TET-3 (**h**) were determined using qRT-PCR. Results expressed as mean ± SD. Statistical significance is indicated with asterisks:*p < 0.05, **p < 0.01, ***p < 0.001 using one-way ANOVA (and non-parametric) test

In order to estimate the replicative cellular senescence, we measured DNA content in ASCs derived from the three age groups in order to provide the information on cell position in the cell cycle. We found that the percentage of ASCs in G1/G0 significantly increased with donors’ age (*p* < 0.01). The result correlated with the decreased number of cells derived from the youngest individuals in the S phase compared with ASC_5–15_ and ASC_15< _ (*p* < 0.01). Interestingly, no significant differences between ASC_5–15_ and ASC_15< _ were observed (Fig. 3a, b). What is more, we performed western blot analysis to determine the phosphorylation of H2AX at serine-139 (known as γH2AX foci), a double-strand breakage (DSB) marker, and protein level of DNA methyltransferase 1 (DNMT1), which catalyzes the DNA methylation. Phosphorylation of γH2AX was highly enhanced in ASC_15< _ compared with the youngest and middle-aged donors (*p* < 0.01) (Fig. 3e). Interestingly, an inverse correlation was observed in the DNMT1 protein level, which is significantly downregulated in ASC_5–15_ and ASC_15< _ (*p* < 0.05 and *p* < 0.01, respectively) (Fig. 3f). On the contrary, we found that the transcript levels of TET-2 and TET-3 were significantly upregulated in ASC_< 5_ compared with the oldest group (*p* < 0.05, *p* < 0.01) (Fig. 3g, h). The ten-eleven translocation (Tet) family is a group of enzymes that catalase DNA demethylation. Furthermore, we observed decreased expression of C-X-C chemokine receptor type 4 (CXCR4) in ASCs from aged donors (Fig. 3d). What is more, western blot analysis showed diminished octamer-binding transcription factor 3/4 (Oct 3/4) protein level in ASC_15< _ compared with the young group (*p* < 0.05) (Fig. 3c). Furthermore, we did not find any significant differences in the 5–15 and < 15 groups. It may suggest that ASC stemness decreased rapidly after the age of 5. In addition, data confirm that increasing donor age is highly correlated with an increased cell number in the G1/G0 stage of the cell cycle, enhanced accumulation of γH2AX foci, and decreased expression of DNMT1.

### Age-related alternations in the expression of apoptosis-related genes

**Fig. 4:**
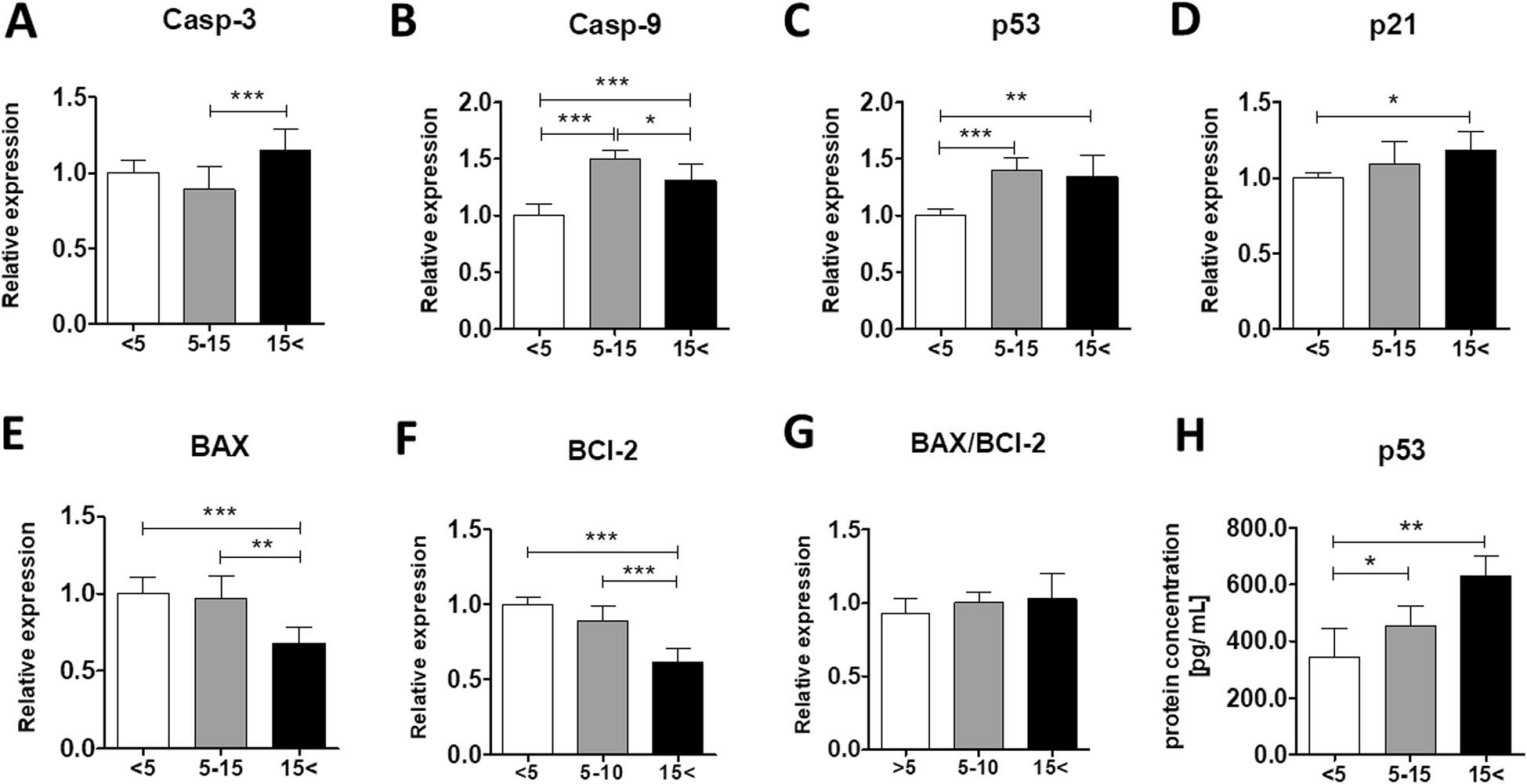
Relative expressions of apoptosis-related genes in ASCs of the three age groups. Transcript levels of Casp-3 (**a**), Casp-9 (**b**), p53 (**c**), p21 (**d**), BAX (**e**), and BCl-2 (**f**) were estimated using the qRT-PCR method. **g** BAX/BCl-2 ratio was determined using the relative expression values of both BAX and BCl-2. **h** Protein levels of p53 were determined with ELISA method. Results expressed as mean ± SD. Statistical significance is indicated with asterisks: *p < 0.05, **p < 0.01, ***p < 0.001 using one-way ANOVA (and non-parametric) test

To determine the expression of apoptosis-related genes, we performed qRT-PCR for Caspase-3 (Casp-3), Casp-9, p53, p21, Bcl-2-associated X protein (BAX), and B cell lymphoma 2 (BCl-2). We observed an age-related increase in the mRNA levels of both caspases. Relative expression of Casp-3 was significantly higher in ASC_< 15_ compared with ASC_5–15_ (*p* < 0.001) (Fig. 4a). Similarly, Casp-9 mRNA levels were significantly upregulated in both ASC_5–15_ and ASC_15< _ in comparison with cells isolated from young horses (*p* < 0.001) (Fig. 4b). Furthermore, the mRNA expression of p53 and p21 significantly increased in ASCs derived from old individuals (*p* < 0.01 and *p* < 0.05, respectively) compared with the young group (Fig. 4c, d). Moreover, the expression of p53 was also significantly enhanced in the middle-aged group in comparison with ASC_< 5_. Similarly, the p53 protein level increased significantly in ASC_5–15_ and ASC_15< _ (*p* < 0.05 and *p* < 0.01, respectively) next to the group of the youngest individuals (Fig. 4h). The BCl-2 family proteins, such as the pro-apoptotic BAX and pro-survival BCl-2, play a key role in the regulation of apoptosis. Interestingly, there were no significant differences in BAX/BCl-2 ratio between the experimental groups (Fig. 4g).

### Age-related changes in oxidative stress in equine ASCs

**Fig. 5:**
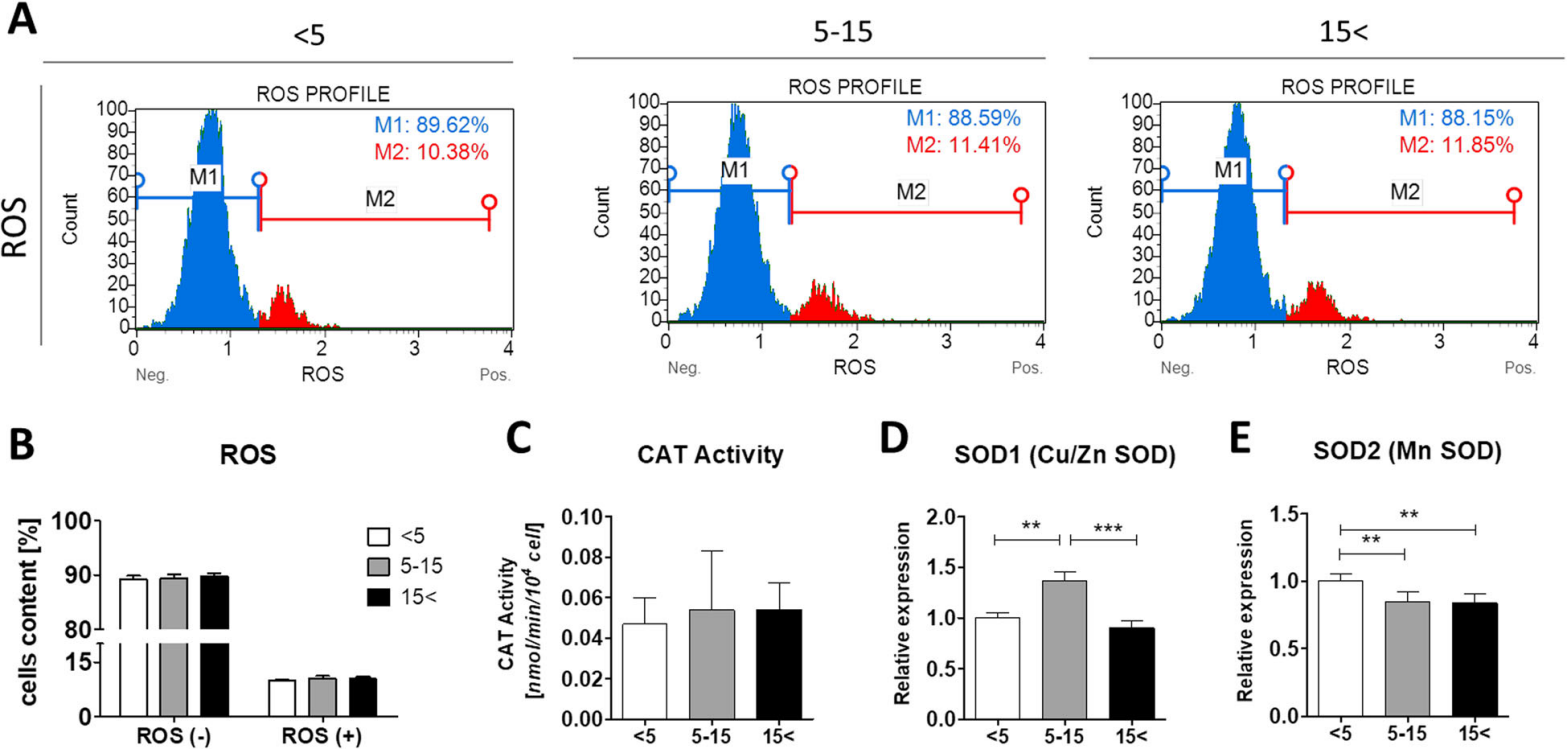
Age-related decline in oxidative stress of ASCs. **a** Evaluation of ROS accumulation in ASCs using Muse™ Cell Analyzer. **b** Percentage of ROS(−) live cells and ROS(+) cells. **c** Catalase activity was detected by Catalase (CAT) Assay Kit. **d**, **e** Transcript levels of SOD 1 (Cu/Zn SOD) and SOD2 (Mn SOD) were determined using the qRT-PCR method. Results expressed as mean ± SD. Statistical significance is indicated with asterisks: *p < 0.05, **p < 0.01, ***p < 0.001 using one-way ANOVA (and non-parametric) test

The aging process is associated with the excessive production of ROS. To assess the intracellular ROS accumulation in ASCs from the three horse age groups, cells were subjected to flow cytometry-based system analysis. Interestingly, we did not detect a significant fluorescence signal of ROS in ASCs from the three age groups (Fig. 5a, b). Similarly, there were no significant differences in catalase (CAT) activity between the experimental groups. Enhanced CAT expression and activity are considered as major oxidative stress markers. CAT, along with superoxidase (SOD) family enzymes, are endogenous antioxidant enzymes. SODs, including cytosolic SOD1 (Cu/Zn SOD) and mitochondrial SOD2 (Mn SOD), are involved in the first step of the antioxidant enzymatic cascade catalyzing the dismutation of superoxide anions into oxygen and hydrogen peroxide (H_2_O_2_), whereas CAT is responsible for H_2_O_2_ detoxification [41]. In order to determine the mRNA levels of SOD1 and SOD2, qRT-PCR was performed. We found that the relative expression of SOD1 is significantly increased in the middle-aged group compared with ASC_< 5_ (*p* < 0.01) and ASC_15< _ (*p* < 0.001) (Fig. 5d). On the contrary, we observed a negative correlation between the expression of SOD2 and animal age. SOD2 is significantly downregulated in both the middle-aged and old groups (*p* < 0.01) (Fig. 5e).

### Increased age alters mitochondria dynamics in EqASCs

**Fig. 6:**
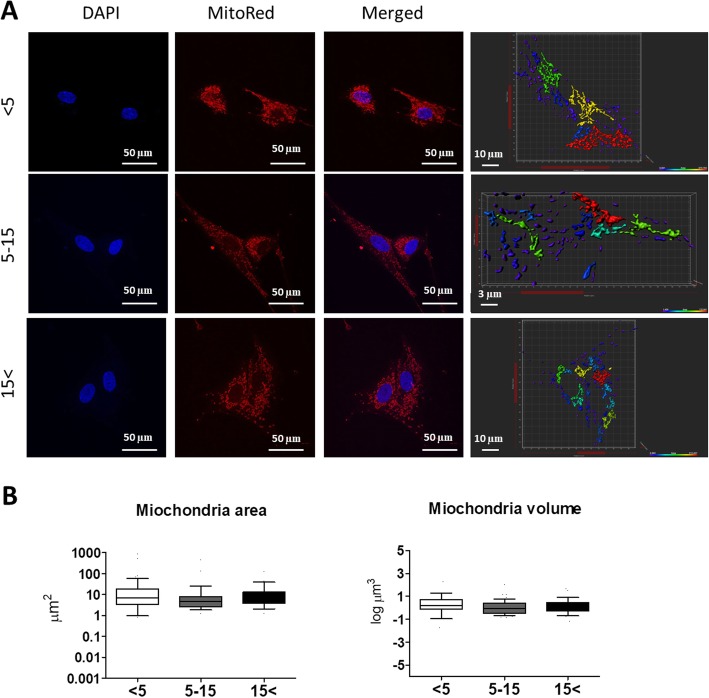
Age-related changes in the mitochondria network. **a** Mitochondria visualization using MitoRed staining (red). Cells’ nuclei were counterstained with DAPI (blue). **b** Mitochondria were analyzed morphometrically to evaluate the mitochondria area and mitochondria volume using Bitplane Imaris software. Results expressed as mean ± SD. Statistical significance is indicated with asterisks: *p < 0.05, **p < 0.01, ***p < 0.001 using one-way ANOVA (and non-parametric) test

**Fig. 7:**
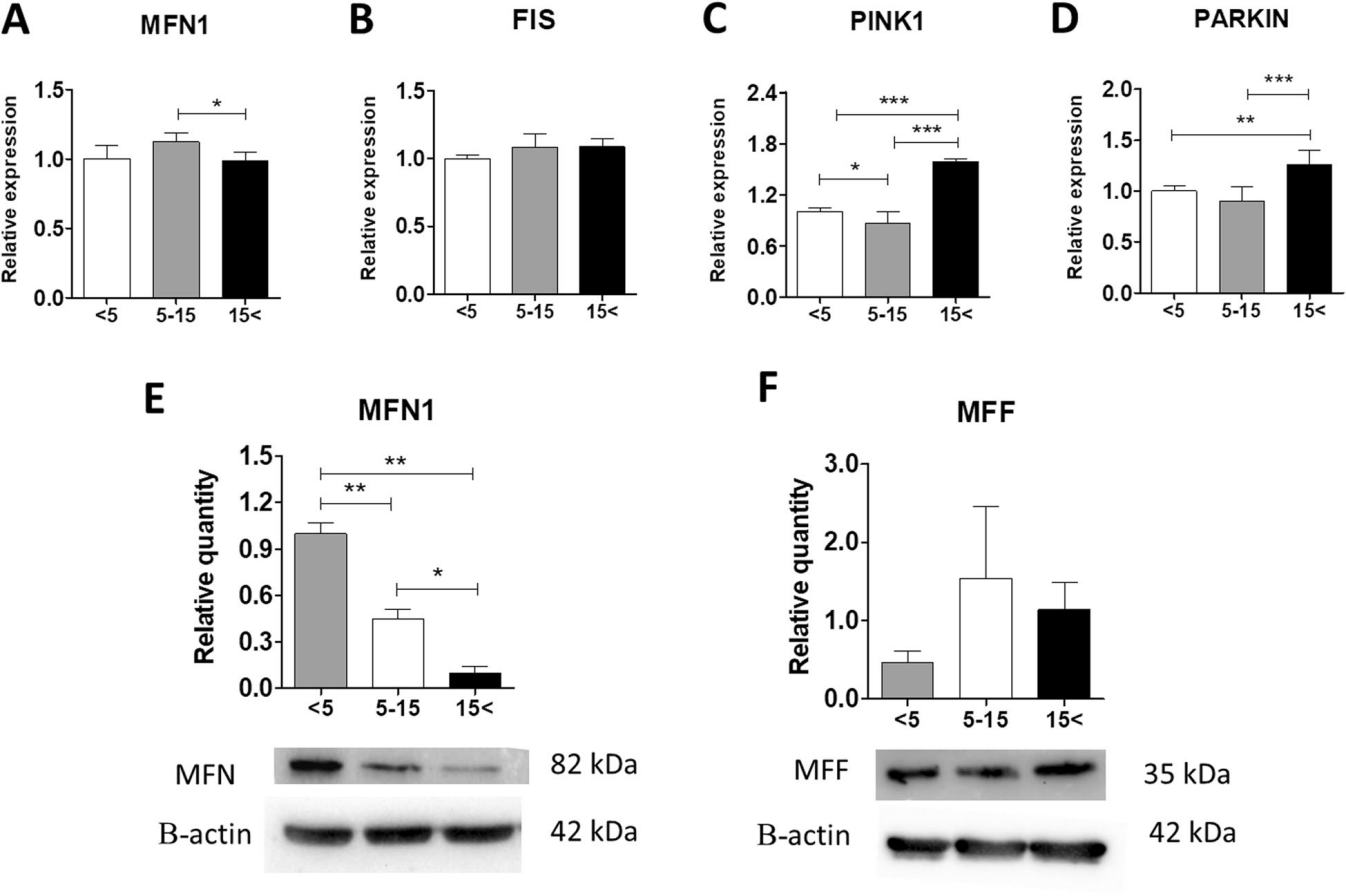
Analysis of mitochondria dynamics in ASCs using qRT-PCR and western blot techniques. Relative mRNA expression of MFN1 (**a**), FIS1 (**b**), PINK1 (**c**), and PARKIN (**d**) determined by qRT-PCR. **e**, **f** Protein levels of MFN1 and MFF were estimated using western blot. Relative quantity was determined using Image Lab software after normalization with β-actin as a loading control. Representative blots are shown in the bottom panels. Results expressed as mean ± SD. Statistical significance is indicated with asterisks: *p < 0.05, **p < 0.01, ***p < 0.001 using one-way ANOVA (and non-parametric) test

Mitochondria are highly dynamic, semi-autonomous organelles undergoing coordinated fusion and fission cycles. Impaired mitochondria function leads to several metabolic disorders. MitoRed staining revealed that mitochondria of ASCs derived from the middle-aged and the oldest groups exhibited fragmented morphology (Fig. 6a). Interestingly, image-based analysis of the mitochondria area and volume with Bitplane Imaris software showed no significant differences between the experimental groups (Fig. 6b). Changes in the expression of mitochondrial dynamics-related markers were evaluated using qRT-PCR and western blot analysis. The analysis showed that both mRNA and protein levels of mitofusin 1 (MFN1), the key regulator of mitochondrial fusion, are significantly decreased in ASCs from the oldest individuals in comparison with ASC_5–15_ (*p* < 0.05) (Fig. 7a, e). Moreover, MFN1 protein expression is upregulated in the youngest group compared with ASC_5–15_ and ASC_15< _ (*p* < 0.01) (Fig. 7e). Interestingly, there were no significant differences in mitochondrial fission 1 (FIS1) mRNA and protein levels (Fig. 7b, f). Moreover, while aged ASCs (ASC_15< _) are characterized by enhanced expression of PTEN-induced kinase (PINK1) (*p* < 0.001), they also exhibited increased mRNA level of parkin RBR E3 ubiquitin-protein ligase (PARKIN) (*p* < 0.01) compared with the youngest group (Fig. 7c, d). In summary, the results from qRT-PCR and western blot analysis suggest enhanced mitochondrial fusion in ASCs from young individuals, but the process decreased with increasing age.

### Age-related pro-inflammatory and anti-inflammatory cytokines and miRNA dysregulation

**Fig. 8:**
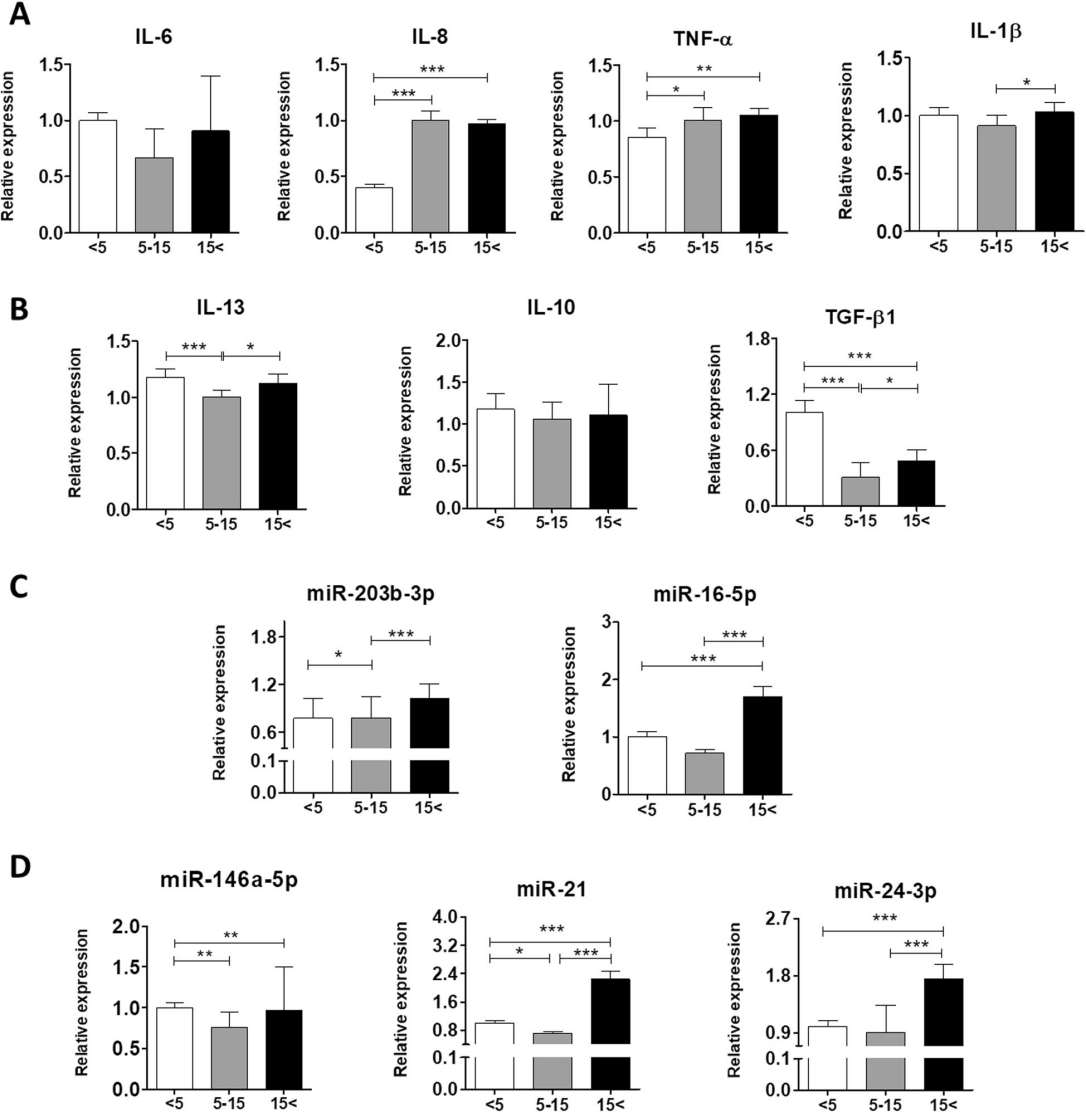
Changes in the expressions of pro- (**a**) and anti-inflammatory (**b**) cytokines. Relative expressions of inflammation-associated miRNA (**c**, **d**). Each analysis was performed using the qRT-PCR method. Results expressed as mean ± SD. Statistical significance is indicated with asterisks: *p < 0.05, **p < 0.01, ***p < 0.001 using one-way ANOVA (and non-parametric) test

**Fig. 9:**
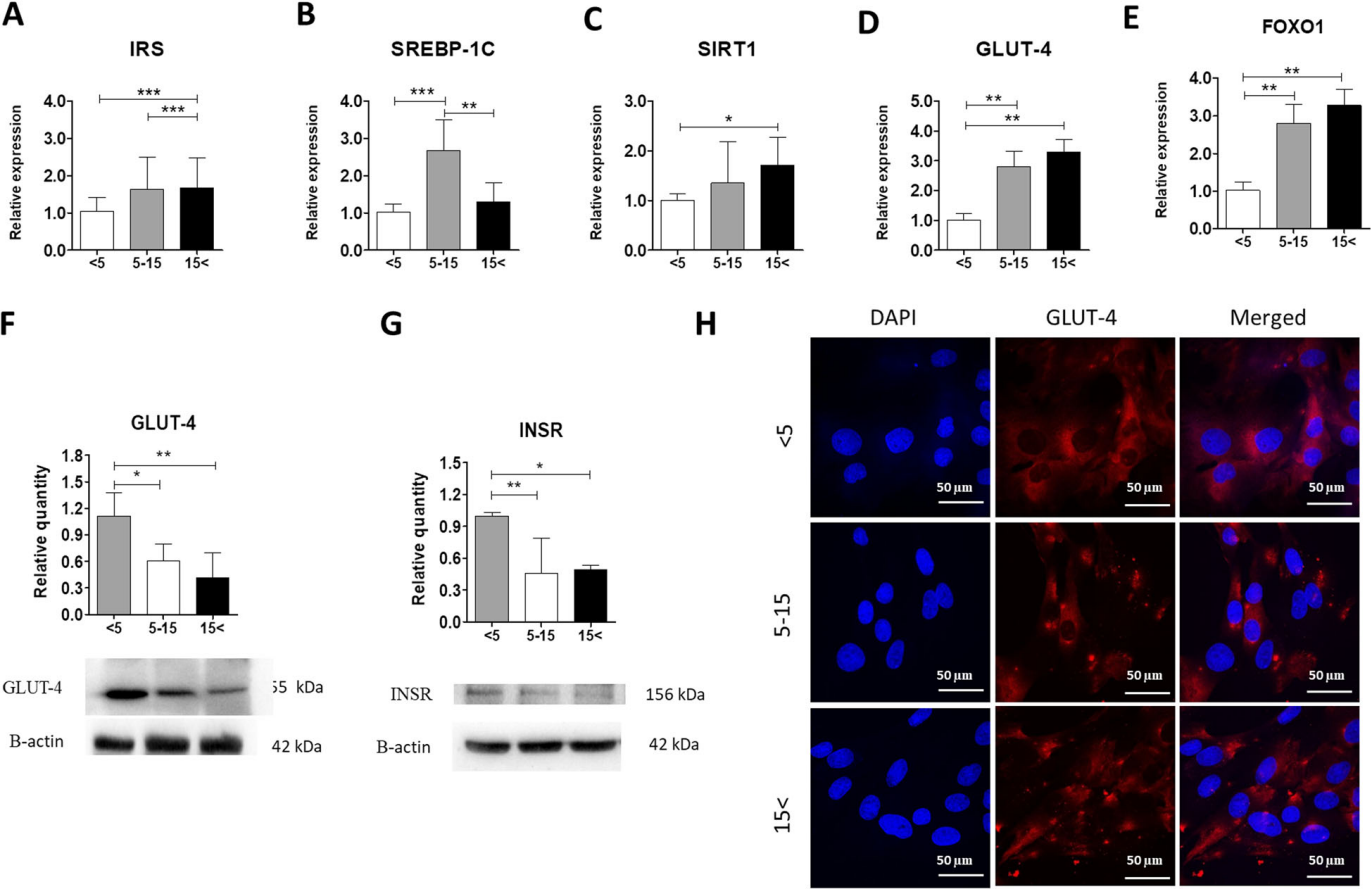
Age-associated alternations in the expressions of insulin resistance-related markers. Relative transcript levels of IRS (**a**), SREBP-1C (**b**), SIRT1 (**c**), GLUT-4 (**d**), and FOXO1 (**e**) were determined using the qRT-PCR technique. Protein contents of GLUT-4 (**f**) and INSR (**g**) were evaluated with the western blot method. β-Actin was used as a loading control. Results expressed as mean ± SD. Statistical significance is indicated with asterisks: *p < 0.05, **p < 0.01, ***p < 0.001 using one-way ANOVA (and non-parametric) test. **h** Representative images of immunofluorescence staining for GLUT-4 in equine ASCs from the three age-matched groups

In order to elucidate the alternations in the expressions of inflammation-related cytokines and miRNA, qRT-PCRs were performed. The analysis revealed that pro-inflammatory cytokines such as IL-8 and TNF-α were significantly upregulated in ASC_5–15_ and ASC_15< _ compared with the youngest group (Fig. 9a). What is more, we observed markedly significant differences in the expression of IL-1β in ASCs derived from the old individuals compared with ASC_5–15_ (*p* < 0.05) (Fig. 8a). On the contrary, the middle-aged and old groups exhibited a significant decrease in mRNA expression of anti-inflammatory transforming growth factor β1 (TGF-β1) versus the youngest group (*p* < 0.001) (Fig. 9b). Interestingly, the mRNA level of IL-13 was significantly reduced in ASC_5–15_ (*p* < 0.001) but increased in the oldest age group (*p* < 0.05) (Fig. 8b). Additionally, we did not observe any significant differences in the expression of another anti-inflammatory cytokine, IL-10. What is more, we decided to estimate the changes in relative expressions of microRNA involved in the regulation of inflammation. qRT-PCR results revealed overexpression of pro-inflammatory miR-203-3p and miR-16 in ASCs derived from aged individuals (Fig. 8c). In addition, levels of miR-146a-5p, miR-21, and miR-24-3p are also increased in the oldest group (Fig. 8d). Interestingly, each of them plays a major role in the anti-inflammatory response.

### Increasing age alters expressions of insulin resistance-related markers

In order to estimate the alternations in the expressions of insulin resistance-related markers, we used qRT-PCR, western blot, and immunofluorescence methods. We observed increased expressions of insulin receptor substrate (IRS) (Fig. 9a), sirtuin 1 (SIRT1) (Fig. 9c), glucose transporter 4 (GLUT-4) (Fig. 9d), and forkhead box protein O1 (FOXO1) (Fig. 9e) in ASC derived from middle-aged and old individuals. The transcript level of sterol regulatory element-binding protein 1C (SREBP-1C), required for lipid synthesis, is significantly upregulated only in ASC_5–15_ (Fig. 9b). Interestingly, using the western blot technique, we found that both protein expression of GLUT-4 (Fig. 9f) and INSR (Fig. 9g) significantly decreased with increasing age. What is more, immunofluorescence staining confirmed the result. ASCs derived from horses older than 5 exhibited a lower level of GLUT-4 (Fig. 9h).

### Age-linked changes in the expressions of UPR-related markers

**Fig. 10:**
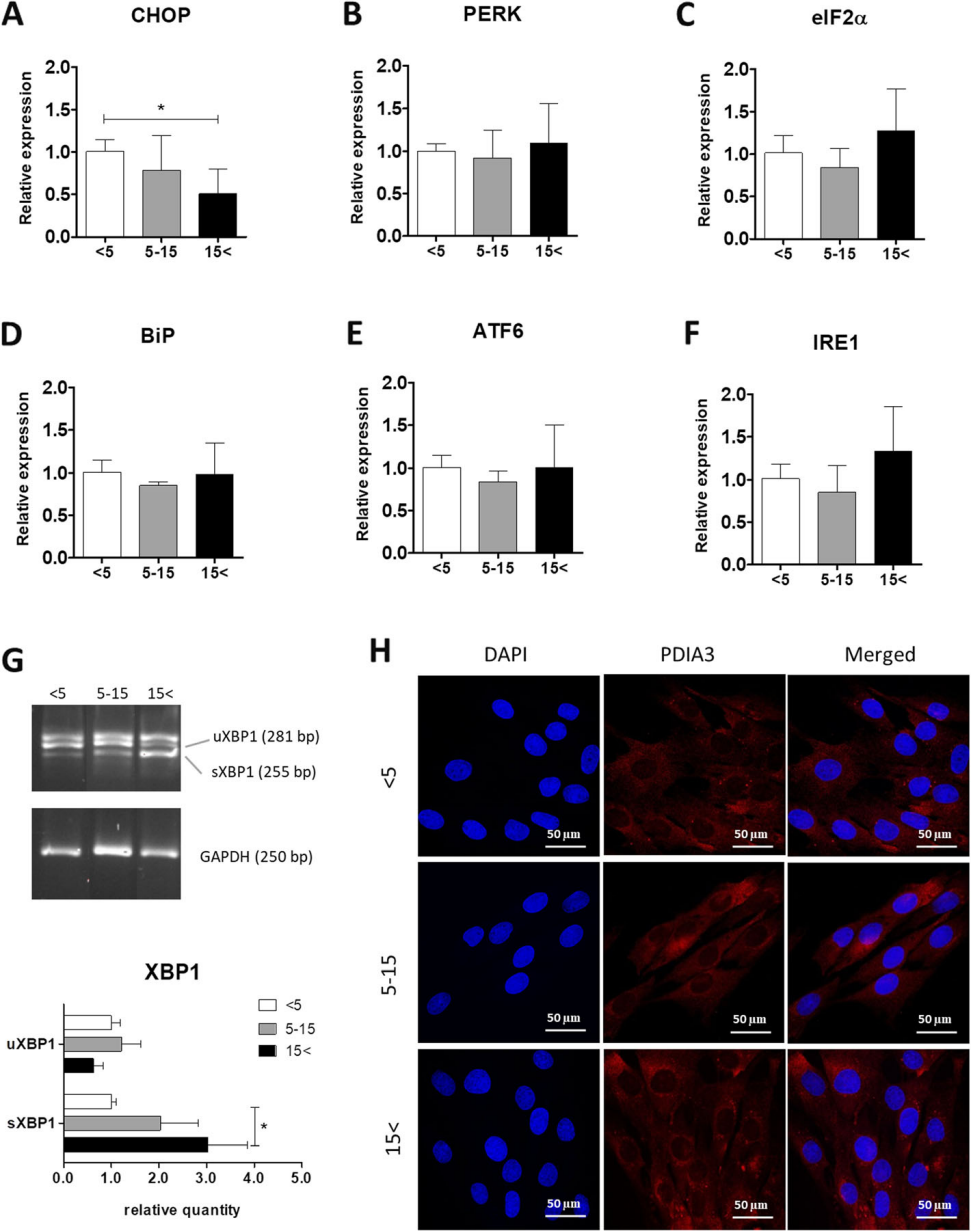
Age-associated changes in the expressions of UPR-linked markers. Expressions of CHOP (**a**), PERK (**b**), eIF2α (**c**), BiP (**d**), ATF6, (**e**), and IRE1 (**f**) were determined using qRT-PCR. **g** To evaluate the mRNA levels of uXBP1 and sXBP1, RT-PCR was performed with XBP1 primers, and the PCR products were run in the 2% agarose gel. Relative quantity of uXBP1 and sXBP1 was determined using Image Lab software after normalization with GAPDH as a reference gene. Results expressed as mean ± SD. Statistical significance is indicated with asterisks: *p < 0.05, **p < 0.01, ***p < 0.001 using one-way ANOVA (and non-parametric) test. **h** Representative confocal microscopy images of PDIA3 staining

To determine whether ASC senescence is associated with ER stress, we analyzed the mRNA levels of UPR-related markers, such as CHOP, PERK, eukaryotic translation initiation factor 2 α (eIF2α), binding immunoglobulin protein (BiP), ATF6, IRE1, uXBP1, and sXBP1. The results showed that there were no significant differences in the expressions of CHOP, PERK, eIF2α, BiP, ATF6, and IRE1 (Fig. 10a–f). On the contrary, the level of sXBP1 was significantly elevated in ASC_15< _ compared with ASC_< 5_ (Fig. 10g). A classical UPR is induced with enhanced expressions of generic chaperones and protein disulfide isomerases (PDI) like PDIA3. In order to determine the protein level of PDIA3 and visualize ER, immunofluorescence staining was performed. Enhanced PDIA3 expression was confirmed by a substantial increase in fluorescence signal in both the middle-aged and the oldest group (Fig. 10h).

## Discussion

Adult stem cells, including ASCs, play a pivotal role in tissue maintenance and its regeneration throughout the lifespan of multicellular organisms [42]. In both, human and veterinary medicine, ASCs are considered as a promising tool to treat a variety of disorders such as bone, cartilage, and spinal cord injury as well as several metabolic disorders. Due to the fact that over 90% of MSCs typically die in the first few days of transplantation, there is a need for a deep and comprehensive analysis of their properties regardless of donor age. Hallmarks of aging, such as metabolic alternations, accumulation of molecular damage, and changes in epigenome stability are partially discovered. However, the detailed molecular changes contributing to mesenchymal stem cell senescence are not fully understood. In the present study, we showed the molecular alternations that may have a pivotal role in the regenerative potential of ASCs isolated from aged horses. Replicative cellular senescence has been described as a progressive loss of proliferative capacity of cells during their repeated replication [43]. Moreover, replicative senescence is highly correlated with cell cycle arrest in the G1/G0 phase, DNA damage, and changes in DNA methylation dynamics. Therefore, we showed that ASCs derived from middle-aged and old horses were characterized by diminished proliferative potential and a higher percentage of cells in the G0/G1 stage of the cell cycle. We also revealed increased levels of miR-101 and miR-17-5p in ASC_15<,_ involving in negative regulation of proliferation and migration. In addition, miR-101 and miR-17-5p enhance apoptosis and senescence in various types of cells including stem cells [44–46]. Moreover, the cells exhibited other molecular features typical of senescence cells, such as increased β-galactosidase activity, accumulation of γH2AX foci, diminished level of DNMT1, and “flattened” morphology. Several studies reported that the excessive accumulation of γH2AX in cell cultures was correlated with DSB induction and telomere erosion in somatic and germ tissues of aging mice. The observations become common events in mammalian aging [47]. In addition, the age-related increase in the accumulation of γH2AX foci in ASC_15< _ may also contribute to the age-associated deficiency of γH2AX de-phosphorylation processes. What is more, replicative senescence-related alternations in the chromatin remodeling process provide an additional source of γH2AX foci [48]. Similarly, the loss of DNMT1 protein is highly correlated with the aging process. Aged tissues show global loss of genomic DNA methylation in all genomic compartments [49]. A study of Bollati et al. in elderly people revealed a gradual age-related loss of genomic DNA methylation within the same patients over an 8-year span [50, 51]. It suggests that the accumulation of genetic alternations, as well as dysregulation of epigenetic signatures, leads to genomic instability and may ultimately cause cellular senescence and apoptosis [47, 52, 53]. Since Tet proteins are expressed in various cell lineages, recent studies showed that Tet proteins are required to maintain mesenchymal stem cell homeostasis [54]. In the present study, we observed the upregulation of TET-2 and TET-3 in ASC_< 5_ compared with the oldest group. Moreover, reduced expression of CXCR4 in ASCs from aged donor groups suggests lower efficiency of homing of ASC_15< _ toward tissue damage [55]. CXCR4 is a chemokine-specific receptor for stromal-derived factor 1 (SDF1) which plays a key role in stem cell survival, proliferation, and migration. Furthermore, CXCR4/SDF1 axis is essential not only for MSCs survival, but also stimulates cytokine production and cell migration. These properties of MSCs become considered as the most important factors for stem cell transplantation [56]. Interestingly, significant downregulation of the Oct3/4 protein level in ASC_15< _ was observed. Several studies revealed that Oct 3/4 confers MSC self-renewal and multipotency, although its level is significantly lower than in embryonic stem cells [57]. These results infer that aged ASCs exhibited reduced expression of stemness-determining genes/proteins. Apoptosis, a programmed cell death, is a highly controlled and regulated mechanism required for normal cell turnover and tissue homeostasis. On the other hand, increased apoptosis in stem cells highly limits tissue regeneration. We showed that increasing donor age is linked to high expression of apoptosis-related genes, such as Casp-3, Casp-9, p53, and p21. Several studies revealed that cellular senescence is a multi-step and highly dynamic process, which involves prolonged inhibition of Cdk-cyclin activity by p21 [58, 59]. Moreover, there is a growing body of evidence that senescence-like phenotype including detectable SA-β-gal activity, transient upregulation of p53 and p21 followed by binucleated morphology, and accumulation of γH2AX foci is associated with cell cycle arrest [59, 60]. Mitochondria are the “power plants” of the eukaryotic cells, and their respiratory chain complex not only provides chemical energy in the form of ATP, but also generate large amounts of ROS. In accordance with Harman’s mitochondrial free radical theory of aging, mitochondria dysfunction is caused by excess ROS accumulation and impaired antioxidant system within the organelle leading to the accumulation of mutations in mtDNA [61]. We observed decreased expression of SOD2 in both ASC_5–15_ ASC_15< _. Deficiency of mitochondrial SOD2 may lead to mitochondrial dysfunction and promote cellular senescence in ASCs from the middle-aged and old animal groups. Mitochondria undergoes coordinated fusion and fission cycles. In addition, they have an inner ability to sense their health condition, and stressed mitochondria activate compensatory quality control mechanism involving degradation of damaged mitochondria (mitophagy) and fission making them an excellent indicator for estimating cell state of health [62]. Changes in mitochondria dynamics and mitochondria membrane potential lead to cellular dysfunction in aged organisms. Moreover, several reports suggest that impaired mitochondrial function is highly linked to age-related diseases like neurodegenerative diseases, cancer, and metabolic disorders [62, 63]. The current study revealed that mitochondria of ASCs derived from the middle-aged and the oldest groups exhibited fragmented morphology. Furthermore, we observed a significant decrease in mRNA and protein levels of MFN whereas upregulation of PINK1 and PARKIN in ASCs derived from aged individuals. Interestingly, no significant differences in the expression of FIS were observed in the experimental groups. Recent studies showed that a similar correlation is observed in the skeletal muscle of old individuals [64, 65]. PINK1 and PARKIN are synergistically involved in a common signaling pathway regulating mitophagy and/or mitochondrial maintenance. The lack of significant differences in the mRNA and protein levels of FIS1 suggests that fission is constant in the three age groups. What is more, mitophagy-related gene expression was increased in ASCs derived from aged horses possibly by reduced mitochondrial fusion. Enhanced expression of PINK1 and PARKIN may suggest that in aged ASCs, mitochondria may undergo adaptation via mitophagy to maintain mitochondrial homeostasis. Senescent cells are also characterized by a stable growth arrest and other phenotypic alternations including upregulation of pro-inflammatory cytokines, which is termed the SASP. We showed that ASCs derived from aged horses exhibited high mRNA levels of IL-8, TNF-α, and IL-1β while low transcript level of anti-inflammatory TGF-β1. Upregulation of pro-inflammatory cytokines, such as TNF-α and IL-6, contributes markedly to the inflammation-aging theory in human healthy elderly individuals and plays a key role in several age-related disorders. On the other hand, several studies reported that higher IL-10 serum levels are observed in elderly people due to its role in the suppression of IL-6, TNF-α, and IL-8 [66]. Our recent findings, along with the data reported by others, showed that TGF-β1 deficiency is associated with disruption of normal stem cell physiology [67, 68]. Our results showed that ASCs from aged donors exhibited pro-inflammatory phenotype typical for senescent cells. Inflammation is a complicated and multi-step pathophysiological process, which regulation is crucial to prevent tissue damage. Therefore, we performed qRT-PCR to determine the expression of microRNA, which was shown to be essential for the regulation of inflammation. We found that ASCs derived from the oldest individuals exhibited significant upregulation of miR-203b-3p and miR-16-5p, which are involved in the pro-inflammatory response and miR-146a-5p, miR-21, and miR-24-3p that act as anti-inflammatory factors. Tian et al. showed that transfection of miR-16 mimics stimulated nuclear translocation of nuclear factor kappa-light-chain-enhancer of activated B cells (NF-κB) p65 protein and promoted the expression of pro-inflammatory cytokines, such as interferon γ (IFN-γ) and IL-8 [69]. The study of Guan et al. provided evidence that miR-146a repress aging-associated osteoarthritis and inflammation-induced post-traumatic osteoarthritis [70]. Moreover, miR-146a reduces the production of IL-1β and IL-6 in B cells by targeting IL-1 receptor-associated kinase 1 (IRAK1) but not TNF receptor-associated factor 6 (TRAF6) [71]. Otherwise, miR-21 reduces the activity of the Toll-like receptor (TLR)/NF-κB signaling pathway via the downregulation of TRAF6 [72]. On the contrary, miR-24 inhibits NF-κB nuclear translocation and biosynthesis of TNF-α and IL-6 [73]. In summary, ASCs from aged horses exhibited upregulation of both anti- and pro-inflammatory miRNAs. Interestingly, anti-inflammatory miRNA did not affect the expressions of pro-inflammatory cytokines. The obtained results suggest that the activity of pro-inflammatory miRNA prevails over anti-inflammatory properties of miR-146a, miR-21, and miR-24-3p. The aging process is also highly correlated with insulin resistance occurrence. In older individuals, age-related rise in the volume of subcutaneous and visceral fat, as well as the accumulation of senescent cells, exhibited an inflammatory phenotype that interferes with the insulin signaling pathway. The impaired insulin sensitivity is caused by dysfunction of the intracellular signaling pathway involving the INSR, IRS, phosphoinositol 3-kinase (PI3K), and protein kinase B (Akt). Akt promotes the translocation of GLUT-4, which moves to the cell surface to transport glucose into the cell [20]. The disturbance leads to abnormal glucose metabolism in metabolically important tissue, such as adipose tissue [74]. Using qRT-PCR and western blot methods, we found that increased transcript levels of IRS, SIRT1, GLUT-4, and FOXO1 are linked to increasing donor age. Excess lipid synthesis and accumulation induces insulin resistance during aging [20]. Studies of Kim and Spiegelman showed that the number of SREBP-1C gene transcripts increased significantly during adipogenic differentiation of pre-adipocyte cell line [75]. On the contrary, FOXO1 activation is highly associated with increased glucose uptake. Su et al. demonstrated that FOXO1 is positively correlated with the expression of pro-inflammatory cytokine, IL-1β, that inhibits insulin signaling cascade [76]. There is a growing body of evidence indicating that the SIRT1 axis plays a dual role in the insulin signaling and senescence process. It has been proposed that the deacetylation mechanism of SIRT1 may reduce the activity of FOXO1 leading to increased glycolysis. Moreover, SIRT1 may be crucial in the phosphorylation and activation of Akt, which promotes the GLUT-4 translocation [77]. SIRT1 negatively regulates the expression of SASP factors, such as IL-6 and IL-8, at the transcriptional level [78]. Furthermore, SIRT1 also deacetylates p53 promoting its degradation. Overexpression of SIRT1 may play a protective role against the expression of senescence-associated factors. Interestingly, the relationship between donor age and protein levels of insulin receptor (INSR) and GLUT-4 is completely different. We showed that protein levels of both GLUT-4 and INSR decreased significantly in ASCs isolated from middle-aged and old individuals. Generally, GLUT-4 in adipose tissue is essential for glucose homeostasis. Leguisamo et al. have provided evidence that insulin resistance is closely related to reduced GLUT-4 content in insulin-sensitive tissue including adipose tissue in a rat model [79]. Low levels of those proteins significantly reduced glucose homeostasis and may lead to insulin resistance. The proper function of the ER is crucial to maintain cellular proteostasis. Considering the vital functions of the ER in ASCs, it might be expected that its dysfunction affects the regenerative capacity of stem cells. In the present study, we showed that ASCs derived from the aged horses are characterized by an increased mRNA level of sXBP1 indicating enhanced IRE1 endonuclease activity. Moreover, IRE1 catalyzes the degradation of several mRNA and microRNAs through a process called regulated IRE1-dependent decay (RIDD), which plays an important role in aging-related processes, such as inflammation and apoptosis. A similar correlation was observed in aged mouse adipose tissue, oocytes, kidney, and muscle, whereas the differences in the expressions of unspliced (281 bp) and spliced XBP1 (255 bp) (uXBP1/sXBP1) forms were not observed in mouse heart and lung and human muscle. Moreover, other authors described no changes in the expression of IRE1 in aged human muscle, mouse heart, and rat brain [25]. XBP1 targets PDIA3, which upregulation was observed in ASC_15< _ suggesting impaired ER protein folding and activation of UPR and apoptosis pathway [80]. Moreover, the study of Zhao et al. revealed that PDIA3 promoted apoptosis through activation of Bak oligomerization and permeabilization of the mitochondrial outer membrane [81]. Therefore, we propose that elevated expressions of sXBP1 and PDIA3 are linked to an increase in the expressions of pro-apoptotic genes.

## Conclusions

Metabolic deterioration and decline in the regenerative potential of tissues are common features of aging. Tissue regeneration is maintained through the presence of multipotent somatic stem cells like ASCs. Here, we showed that the most important factors for ASC transplantation such as survival potential, proliferation activity, and expressions of genes involved in stem cell homeostasis and DNA methylation dynamics (DNMT1, TET-2, TET-3, CXCR4, Oct 3/4) significantly decrease in horses over 5 years of age. Moreover, they exhibited senescence-like phenotype (increased β-galactosidase activity, G1/G0 cell cycle arrest, accumulation of γH2AX foci), as well as decreased mitofusion. Similarly to mature, differentiated cells, ASCs derived from aged horses displayed overexpression of pro-inflammatory cytokines and miRNA, whereas downregulation of anti-inflammatory TGFβ1. Interestingly, we showed that protein expressions of GLUT-4 and INSR, as well as cell surface level of GLUT-4, decreased with increasing age. Besides, increasing age significantly enhanced levels of PDIA3 and spliced XBP1, which suggests increased activity of IRE1 branch. Therefore, we conclude that increasing age (> 5) rapidly reduces the major functions of ASCs, such as cell survival, homeostasis, and proliferation activity. These findings have important implications for understanding the molecular alternations in ASCs derived from middle-aged and old horses to find the molecular target for exploring novel therapy improving stem cell homeostasis and regenerative potential.

## Supplementary information

**Additional file 1.** Sequences of primers used in qRT-PCR.

## Data Availability

All datasets generated and analyzed during the study are presented in the manuscript. The accompanying source data and supplementary information are available from the corresponding author upon reasonable request.

## Abbreviations

Akt: Protein kinase B
ASCs: Adipose-derived stem cells
ATF6: Activating transcription factor 6
BAX: BCl-2-associated X protein
BCl-2: B cell lymphoma 2
BiP: Binding immunoglobulin protein
CAT: Catalase
CHOP: CCAAT-enhancer-binding protein homologous protein
CXCR4: C-X-C chemokine receptor type 4
DAPI: 4′,6-Diamidino-2-phenylindole
DMEM: Dulbecco’s modified Eagle’s medium
DNMT1: DNA methyltransferase 1
DPSCs: Dental pulp stem cells
eIF2a: Eukaryotic translation initiation factor 2α
ER: Endoplasmic reticulum
ERAD: ER-associated degradation
FBS: Fetal bovine serum
FIS1: Mitochondrial fission 1 molecule
FOXO1: Forkhead box protein O1
GAPDH: Glyceraldehyde-3-phosphate dehydrogenase
GLUT-4: Glucose transporter 4
IFN-γ: Interferon γ
INSR: Insulin receptor
IRAK1: IL-1 receptor-associated kinase 1
IRE-1: Elevated inositol*-*requiring enzyme 1
IRS: Insulin receptor substrate
MFF: Mitochondrial fission factor
MFN1: Mitofusin 1
MSCs: Mesenchymal stem cells
mtDNA: Mitochondrial DNA
NF-κB: Nuclear factor kappa-light-chain-enhancer of activated B cells
Oct 3/4: Octamer transcription factor ¾
p21: Cyclin-dependent kinase inhibitor 1
PARKIN: Parkin RBR E3 ubiquitin-protein ligase
PBS: Phosphate-buffered saline
PDIA3: Protein disulfide-isomerase A3
PERK: Protein kinase RNA-like endoplasmic reticulum kinase
PFA: Paraformaldehyde
PI3K: Phosphoinositol 3-kinase
PINK1: PTEN-induced putative kinase 1
PVDF: Polyvinylidene difluoride
qRT-PCR: Quantitative reverse transcription polymerase chain reaction
RIDD: IRE1-dependent decay
RONS: Reactive oxygen and nitrogen species
ROS: Reactive oxygen species
SASP: Senescence-associated secretory phenotype
SA-β-gal: Senescence-associated β-galactosidase
SDF1: Stromal-derived factor 1
SEM: Scanning electron microscopy
SIRT1: Sirtuin 1
SOD: Superoxide dismutase
SREBP-1C: Sterol regulatory element-binding transcription factor 1
TET: Ten-eleven translocation methylcytosine dioxygenase
TGF-β1: Transforming growth factor β1
TLR: Toll-like receptor
TNF-α: Tumor necrosis factor α
TRAF6: TNF receptor-associated factor 6
UPR: Unfolded protein response
WJ-MSCs: Wharton’s jelly mesenchymal stem cells
XBP1: X-box binding protein 1

## References

[1] Friedenstein AJ, Chailakhjan RK, Lalykina KS (1970). The development of fibroblast colonies in monolayer cultures of guinea-pig bone marrow and spleen cells. Cell Tissue Kinet.

[2] Ren H, Sang Y, Zhang F, Liu Z, Qi N, Chen Y. Comparative analysis of human mesenchymal stem cells from umbilical cord, dental pulp, and menstrual blood as sources for cell therapy. Stem Cells Int. 2016;2016 Available from: https://www.ncbi.nlm.nih.gov/pmc/articles/PMC4736971/. [cited 2019 Mar 31]. 10.1155/2016/3516574 PMC4736971 26880954

[3] Almalki SG, Agrawal DK (2016). Key transcription factors in the differentiation of mesenchymal stem cells. Differentiation..

[4] Matic I, Antunovic M, Brkic S, Josipovic P, Mihalic KC, Karlak I (2016). Expression of OCT-4 and SOX-2 in bone marrow-derived human mesenchymal stem cells during osteogenic differentiation. Open Access Maced J Med Sci.

[5] Leijs MJC, van Buul GM, Lubberts E, Bos PK, Verhaar JAN, Hoogduijn MJ, et al. Effect of arthritic synovial fluids on the expression of immunomodulatory factors by mesenchymal stem cells: an explorative in vitro study. Front Immunol. 2012;3 Available from: http://journal.frontiersin.org/article/10.3389/fimmu.2012.00231/abstract. [cited 2019 Apr 1]. 10.3389/fimmu.2012.00231 PMC3410447 22876244

[6] Fiorina P, Jurewicz M, Augello A, Vergani A, Dada S, La Rosa S (2009). Immunomodulatory function of bone marrow-derived mesenchymal stem cells in experimental autoimmune type 1 diabetes. J Immunol.

[7] Gnecchi M, He H, Liang OD, Melo LG, Morello F, Mu H (2005). Paracrine action accounts for marked protection of ischemic heart by Akt-modified mesenchymal stem cells. Nat Med.

[8] Biancone L, Bruno S, Deregibus MC, Tetta C, Camussi G (2012). Therapeutic potential of mesenchymal stem cell-derived microvesicles. Nephrol Dial Transplant.

[9] Alves H, Munoz-Najar U, De Wit J, Renard AJS, Hoeijmakers JHJ, Sedivy JM (2010). A link between the accumulation of DNA damage and loss of multi-potency of human mesenchymal stromal cells. J Cell Mol Med.

[10] Kornicka K, Marycz K, Tomaszewski KA, Marędziak M, Śmieszek A. The effect of age on osteogenic and adipogenic differentiation potential of human adipose derived stromal stem cells (hASCs) and the impact of stress factors in the course of the differentiation process. Oxid Med Cell Longev. 2015;2015 Available from: https://www.ncbi.nlm.nih.gov/pmc/articles/PMC4515302/. [cited 2019 Jan 4]. 10.1155/2015/309169 PMC4515302 26246868

[11] Alicka M, Marycz K. The effect of chronic inflammation and oxidative and endoplasmic reticulum stress in the course of metabolic syndrome and its therapy. Stem Cells Int. 2018; Available from: https://www.hindawi.com/journals/sci/2018/4274361/cta/. [cited 2018 Dec 27]. 10.1155/2018/4274361 PMC6217741 30425746

[12] Marycz K, Kornicka K, Basinska K, Czyrek A (2016). Equine metabolic syndrome affects viability, senescence, and stress factors of equine adipose-derived mesenchymal stromal stem cells: new insight into EqASCs isolated from EMS horses in the context of their aging. Oxidative Med Cell Longev.

[13] Marycz K, Kornicka K, Grzesiak J, Śmieszek A, Szłapka J (2016). Macroautophagy and selective Mitophagy ameliorate chondrogenic differentiation potential in adipose stem cells of equine metabolic syndrome: new findings in the field of progenitor cells differentiation. Oxidative Med Cell Longev.

[14] Ali F, Aziz F, Wajid N (2017). Effect of type 2 diabetic serum on the behavior of Wharton’s jelly-derived mesenchymal stem cells in vitro. Chronic Dis Transl Med.

[15] Malaquin N, Martinez A, Rodier F (2016). Keeping the senescence secretome under control: molecular reins on the senescence-associated secretory phenotype. Exp Gerontol.

[16] Starr ME, Saito M, Evers BM, Saito H (2015). Age-associated increase in cytokine production during systemic inflammation-II: the role of IL-1β in age-dependent IL-6 upregulation in adipose tissue. J Gerontol A Biol Sci Med Sci.

[17] Morin CL, Pagliassotti MJ, Windmiller D, Eckel RH (1997). Adipose tissue-derived tumor necrosis factor-alpha activity is elevated in older rats. J Gerontol A Biol Sci Med Sci.

[18] Gao D, Madi M, Ding C, Fok M, Steele T, Ford C (2014). Interleukin-1β mediates macrophage-induced impairment of insulin signaling in human primary adipocytes. Am J Physiol Endocrinol Metab.

[19] Rotter V, Nagaev I, Smith U (2003). Interleukin-6 (IL-6) induces insulin resistance in 3T3-L1 adipocytes and is, like IL-8 and tumor necrosis factor-alpha, overexpressed in human fat cells from insulin-resistant subjects. J Biol Chem.

[20] Park MH, Kim DH, Lee EK, Kim ND, Im DS, Lee J (2014). Age-related inflammation and insulin resistance: a review of their intricate interdependency. Arch Pharm Res.

[21] Khansari N, Shakiba Y, Mahmoudi M (2009). Chronic inflammation and oxidative stress as a major cause of age-related diseases and cancer. Recent Patents Inflamm Allergy Drug Discov.

[22] Liguori I, Russo G, Curcio F, Bulli G, Aran L, Della-Morte D (2018). Oxidative stress, aging, and diseases. Clin Interv Aging.

[23] Zhao G, Cao K, Xu C, Sun A, Lu W, Zheng Y (2017). Crosstalk between mitochondrial fission and oxidative stress in paraquat-induced apoptosis in mouse alveolar type II cells. Int J Biol Sci.

[24] Marycz Krzysztof, Kornicka Katarzyna, Szlapka-Kosarzewska Jolanta, Weiss Christine (2018). Excessive Endoplasmic Reticulum Stress Correlates with Impaired Mitochondrial Dynamics, Mitophagy and Apoptosis, in Liver and Adipose Tissue, but Not in Muscles in EMS Horses. International Journal of Molecular Sciences.

[25] Martínez G, Duran-Aniotz C, Cabral-Miranda F, Vivar JP, Hetz C (2017). Endoplasmic reticulum proteostasis impairment in aging. Aging Cell.

[26] Kitamura M (2008). Endoplasmic reticulum stress and unfolded protein response in renal pathophysiology: Janus faces. Am J Physiol Renal Physiol.

[27] Tabas I, Ron D (2011). Integrating the mechanisms of apoptosis induced by endoplasmic reticulum stress. Nat Cell Biol.

[28] Childs BG, Durik M, Baker DJ, van Deursen JM (2015). Cellular senescence in aging and age-related disease: from mechanisms to therapy. Nat Med.

[29] Dimri GP, Lee X, Basile G, Acosta M, Scott G, Roskelley C (1995). A biomarker that identifies senescent human cells in culture and in aging skin in vivo. Proc Natl Acad Sci U S A.

[30] Search of: mesenchymal stem cells - List Results - ClinicalTrials.gov. Available from: https://www.clinicaltrials.gov/ct2/results?cond=&term=mesenchymal+stem+cells&cntry=&state=&city=&dist=. [cited 2019 Mar 31]

[31] Esteves CL, Sheldrake TA, Mesquita SP, Pesántez JJ, Menghini T, Dawson L (2017). Isolation and characterization of equine native MSC populations. Stem Cell Res Ther.

[32] Ankrum JA, Ong JF, Karp JM (2014). Mesenchymal stem cells: immune evasive, not immune privileged. Nat Biotechnol.

[33] Owens SD, Kol A, Walker NJ, Borjesson DL. Allogeneic mesenchymal stem cell treatment induces specific alloantibodies in horses. Stem Cells Int. 2016;2016 Available from: https://www.ncbi.nlm.nih.gov/pmc/articles/PMC5018342/. [cited 2019 Apr 1]. 10.1155/2016/5830103 PMC5018342 27648075

[34] Marycz K, Weiss C, Śmieszek A, Kornicka K (2018). Evaluation of oxidative stress and mitophagy during adipogenic differentiation of adipose-derived stem cells isolated from equine metabolic syndrome (EMS) horses. Stem Cells Int.

[35] Doubling Time - Online computing with 2 points. Available from: http://www.doubling-time.com/compute.php. [cited 2018 Oct 3]

[36] Chomczynski P, Sacchi N (1987). Single-step method of RNA isolation by acid guanidinium thiocyanate-phenol-chloroform extraction. Anal Biochem..

[37] Livak KJ, Schmittgen TD (2001). Analysis of relative gene expression data using real-time quantitative PCR and the 2^−ΔΔCT^ method. Methods..

[38] Cassimeris L, Engiles JB, Galantino-Homer H. Detection of endoplasmic reticulum stress and the unfolded protein response in naturally-occurring endocrinopathic equine laminitis. BMC Vet Res. 2019;15 Available from: https://www.ncbi.nlm.nih.gov/pmc/articles/PMC6327420/. [cited 2019 May 30]. 10.1186/s12917-018-1748-x PMC6327420 30630474

[39] Barkholt L, Flory E, Jekerle V, Lucas-Samuel S, Ahnert P, Bisset L (2013). Risk of tumorigenicity in mesenchymal stromal cell–based therapies—bridging scientific observations and regulatory viewpoints. Cytotherapy..

[40] Zhao H, Darzynkiewicz Z (2013). Biomarkers of cell senescence assessed by imaging cytometry. Methods Mol Biol.

[41] Stier A, Schull Q, Bize P, Lefol E, Haussmann M, Roussel D (2019). Oxidative stress and mitochondrial responses to stress exposure suggest that king penguins are naturally equipped to resist stress. Sci Rep.

[42] Ermolaeva M, Neri F, Ori A, Rudolph KL (2018). Cellular and epigenetic drivers of stem cell ageing. Nat Rev Mol Cell Biol.

[43] Kim MK, Lee W, Yoon G-H, Chang E-J, Choi S-C, Kim SW (2019). Links between accelerated replicative cellular senescence and down-regulation of SPHK1 transcription. BMB Rep.

[44] Chen L-G, Xia Y-J, Cui Y (2017). Upregulation of miR-101 enhances the cytotoxic effect of anticancer drugs through inhibition of colon cancer cell proliferation. Oncol Rep.

[45] Seeger FH, Zeiher AM, Dimmeler S (2013). MicroRNAs in stem cell function and regenerative therapy of the heart. Arterioscler Thromb Vascular Biol.

[46] Wang X, Li Z, Bai J, Song W, Zhang F (2019). miR-17-5p regulates the proliferation and apoptosis of human trabecular meshwork cells by targeting phosphatase and tensin homolog. Mol Med Rep.

[47] Mah L-J, El-Osta A, Karagiannis TC (2010). γH2AX as a molecular marker of aging and disease. Epigenetics..

[48] Pustovalova M, Grekhova A, Astrelina T, Nikitina V, Dobrovolskaya E, Suchkova Y (2016). Accumulation of spontaneous γH2AX foci in long-term cultured mesenchymal stromal cells. Aging (Albany).

[49] Jung H-J, Byun H-O, Jee BA, Min S, Jeoun U, Lee Y-K (2017). The ubiquitin-like with PHD and ring finger domains 1 (UHRF1)/DNA methyltransferase 1 (DNMT1) axis is a primary regulator of cell senescence. J Biol Chem.

[50] Liu L, van Groen T, Kadish I, Li Y, Wang D, James SR (2011). Insufficient DNA methylation affects healthy aging and promotes age-related health problems. Clin Epigenetics.

[51] Bollati V, Schwartz J, Wright R, Litonjua A, Tarantini L, Suh H (2009). Decline in genomic DNA methylation through aging in a cohort of elderly subjects. Mech Ageing Dev.

[52] Sinclair DA, Oberdoerffer P (2009). The ageing epigenome: damaged beyond repair?. Ageing Res Rev.

[53] Ciccarone F, Tagliatesta S, Caiafa P, Zampieri M (2018). DNA methylation dynamics in aging: how far are we from understanding the mechanisms?. Mech Ageing Dev.

[54] Yang R, Yu T, Kou X, Gao X, Chen C, Liu D (2018). Tet1 and Tet2 maintain mesenchymal stem cell homeostasis via demethylation of the P2rX7 promoter. Nat Commun.

[55] Yang J-X, Zhang N, Wang H-W, Gao P, Yang Q-P, Wen Q-P (2015). CXCR4 receptor overexpression in mesenchymal stem cells facilitates treatment of acute lung injury in rats. J Biol Chem.

[56] Liu X, Duan B, Cheng Z, Jia X, Mao L, Fu H (2011). SDF-1/CXCR4 axis modulates bone marrow mesenchymal stem cell apoptosis, migration and cytokine secretion. Protein & Cell.

[57] Yannarelli G, Pacienza N, Montanari S, Santa-Cruz D, Viswanathan S, Keating A (2017). OCT4 expression mediates partial cardiomyocyte reprogramming of mesenchymal stromal cells. PLoS One.

[58] Stein GH, Drullinger LF, Soulard A, Dulić V (1999). Differential roles for cyclin-dependent kinase inhibitors p21 and p16 in the mechanisms of senescence and differentiation in human fibroblasts. Mol Cell Biol.

[59] Kim YY, Jee HJ, Um J-H, Kim YM, Bae SS, Yun J (2017). Cooperation between p21 and Akt is required for p53-dependent cellular senescence. Aging Cell.

[60] De Santis PM, Gonzalez L, Ascenzi S, Cundari E, Degrassi F (2015). Tetraploid cells produced by absence of substrate adhesion during cytokinesis are limited in their proliferation and enter senescence after DNA replication. Cell Cycle.

[61] Ahlqvist KJ, Suomalainen A, Hämäläinen RH (2015). Stem cells, mitochondria and aging. Biochimica et Biophysica Acta (BBA) - Bioenergetics.

[62] Zahedi A, On V, Phandthong R, Chaili A, Remark G, Bhanu B (2018). Deep analysis of mitochondria and cell health using machine learning. Sci Rep.

[63] Madreiter-Sokolowski CT, Sokolowski AA, Waldeck-Weiermair M, Malli R, Graier WF. Targeting mitochondria to counteract age-related cellular dysfunction. Genes (Basel). 2018;9 Available from: https://www.ncbi.nlm.nih.gov/pmc/articles/PMC5867886/. [cited 2019 Jul 31]. 10.3390/genes9030165 PMC5867886 29547561

[64] Yeo D, Kang C, Gomez-Cabrera MC, Vina J, Ji LL (2019). Intensified mitophagy in skeletal muscle with aging is downregulated by PGC-1alpha overexpression in vivo. Free Radic Biol Med.

[65] Carter HN, Kim Y, Erlich AT, Zarrin-Khat D, Hood DA (2018). Autophagy and mitophagy flux in young and aged skeletal muscle following chronic contractile activity. J Physiol (Lond).

[66] Rea IM, Gibson DS, McGilligan V, McNerlan SE, Alexander HD, Ross OA. Age and age-related diseases: role of inflammation triggers and cytokines. Front Immunol. 2018;9 Available from: https://www.ncbi.nlm.nih.gov/pmc/articles/PMC5900450/. [cited 2019 Aug 5]. 10.3389/fimmu.2018.00586 PMC5900450 29686666

[67] Xu X, Zheng L, Yuan Q, Zhen G, Crane JL, Zhou X (2018). Transforming growth factor-β in stem cells and tissue homeostasis. Bone Res.

[68] Alicka M, Major P, Wysocki M, Marycz K (2019). Adipose-derived mesenchymal stem cells isolated from patients with type 2 diabetes show reduced “stemness” through an altered Secretome profile, impaired anti-oxidative protection, and mitochondrial dynamics deterioration. J Clin Med.

[69] Tian T, Zhou Y, Feng X, Ye S, Wang H, Wu W (2016). MicroRNA-16 is putatively involved in the NF-κB pathway regulation in ulcerative colitis through adenosine A2a receptor (A2aAR) mRNA targeting. Sci Rep.

[70] Guan Y-J, Li J, Yang X, Du S, Ding J, Gao Y (2018). Evidence that miR-146a attenuates aging- and trauma-induced osteoarthritis by inhibiting Notch1, IL-6, and IL-1 mediated catabolism. Aging Cell.

[71] Jiang S, Hu Y, Deng S, Deng J, Yu X, Huang G (2018). miR-146a regulates inflammatory cytokine production in Porphyromonas gingivalis lipopolysaccharide-stimulated B cells by targeting IRAK1 but not TRAF6. Biochim Biophys Acta Mol Basis Dis.

[72] Nara K, Kawashima N, Noda S, Fujii M, Hashimoto K, Tazawa K (2019). Anti-inflammatory roles of microRNA 21 in lipopolysaccharide-stimulated human dental pulp cells. J Cell Physiol.

[73] Tahamtan A, Teymoori-Rad M, Nakstad B, Salimi V. Anti-inflammatory microRNAs and their potential for inflammatory diseases treatment. Front Immunol. 2018;9 Available from: https://www.ncbi.nlm.nih.gov/pmc/articles/PMC6026627/. [cited 2019 Aug 5]. 10.3389/fimmu.2018.01377 PMC6026627 29988529

[74] Barzilai N, Ferrucci L (2012). Insulin resistance and aging: a cause or a protective response?. J Gerontol A Biol Sci Med Sci.

[75] Kim JB, Spiegelman BM (1996). ADD1/SREBP1 promotes adipocyte differentiation and gene expression linked to fatty acid metabolism. Genes Dev.

[76] Su D, Coudriet GM, Hyun Kim D, Lu Y, Perdomo G, Qu S (2009). FoxO1 links insulin resistance to proinflammatory cytokine IL-1 production in macrophages. Diabetes..

[77] Sin TK, Yung BY, Siu PM (2015). Modulation of SIRT1-Foxo1 signaling axis by resveratrol: implications in skeletal muscle aging and insulin resistance. CPB..

[78] Hayakawa T, Iwai M, Aoki S, Takimoto K, Maruyama M, Maruyama W (2015). SIRT1 suppresses the senescence-associated secretory phenotype through epigenetic gene regulation. PLoS One.

[79] Leguisamo NM, Lehnen AM, Machado UF, Okamoto MM, Markoski MM, Pinto GH (2012). GLUT4 content decreases along with insulin resistance and high levels of inflammatory markers in rats with metabolic syndrome. Cardiovasc Diabetol.

[80] Chen D, Wang Y, Chin ER. Activation of the endoplasmic reticulum stress response in skeletal muscle of G93A*SOD1 amyotrophic lateral sclerosis mice. Front Cell Neurosci. 2015;9 Available from: https://www.frontiersin.org/articles/10.3389/fncel.2015.00170/full. [cited 2019 Aug 12]. 10.3389/fncel.2015.00170 PMC4435075 26041991

[81] Zhao G, Lu H, Li C (2015). Proapoptotic activities of protein disulfide isomerase (PDI) and PDIA3 protein, a role of the Bcl-2 protein Bak. J Biol Chem.

