# Supplementary material for: Age-dependent impairment of adipose-derived stem cells isolated from horses
Source: Stem Cell Res Ther. 2020 Jan 3;11:4. doi: 10.1186/s13287-019-1512-6 (PMC6942290; doi:10.1186/s13287-019-1512-6)
Supplement: Supplementary file 1 — Additional file 1. Sequences of primers used in qRT-PCR. [file 13287_2019_1512_MOESM1_ESM.docx]

Additional file 1 Sequences of primers used in qRT-PCR

| **Gene** | **Direction** | **Primer sequence (5’ → 3’)** | **Product size (bp)** | **Accession No.** |
| --- | --- | --- | --- | --- |
| TET-2 | F | ATCCTGATCCTGGTGTGGGA | 143 | XM_023636796.1 |
|  | R | CCTTGACAGGCACAGGTTCT |  |  |
| TET-3 | F | CAGCCTGCATGGACTTCTGT | 188 | XM_023618871.1 |
|  | R | GTTCTCCTCACTGCCGAACT |  |  |
| CXCR4 | F | CAGGTAGCAAAGTGACTCCGA | 138 | XM_005601469.3 |
|  | R | TCATAATCCCCAGAGCCCAC |  |  |
| Casp-3 | F | GGCAGACTTCCTGTATGCGT | 167 | XM_023630401.1 |
|  | R | CCATGGCTACCTTGCGGTTA |  |  |
| p53 | F | TACTCCCCTGCCCTCAACAA | 252 | U37120.1 |
|  | R | AGGAATCAGGGCCTTGAGGA |  |  |
| p21 | F | GAAGAGAAACCCCCAGCTCC | 241 | XM_023633887.1 |
|  | R | TGACTGCATCAAACCCCACA |  |  |
| Casp-9 | F | TCCTACTCCACCTTCCCAGG | 150 | XM_005607504.3 |
|  | R | CTCCGAAACAGCGTGAGCTA |  |  |
| BAX | F | TTCCGACGGCAACTTCAACT | 204 | XM_023650077.1 |
|  | R | GGTGACCCAAAGTCGGAGAG |  |  |
| BCl-2 | F | TTCTTTGAGTTCGGTGGGGT | 164 | XM_014843802.1 |
|  | R | GGGCCGTACAGTTCCACAA |  |  |
| IRS | F | CTGCTGGGGGTTTGGAGAAT | 173 | XM_023642446.1 |
|  | R | TAAATCCTCACTGGAGCGGC |  |  |
| SREBP-1C | F | TCAGCGAGGCGGCTTTGGAGCAG | 80 | XM_008542859.1 |
|  | R | CATGTCTTCGATGTCGGTCAG |  |  |
| SIRT1 | F | ACCAACGGTTTTCATTCTTGTGA | 139 | XM_023643979.1 |
|  | R | TTCGAGGATCTGTGCCAATCA |  |  |
| GLUT-4 | F | CGGGTTTTCAACAGATCGGC | 146 | NM_001081866.2 |
|  | R | CACCTTCTGTGGGGCATTGA |  |  |
| FOXO1 | F | ATTGAGCGCTTGGACTGTGA | 311 | XM_023621529.1 |
|  | R | CGCTGCCAAGTTTGACGAAA |  |  |
| MFN1 | F | AAGTGGCATTTTTCGGCAGG | 217 | XM 001495170.5 |
|  | R | TCCATATGAAGGGCATGGGC |  |  |
| FIS1 | F | GGTGCGAAGCAAGTACAACG | 118 | XM 001504462.4 |
|  | R | GTTGCCCACAGCCAGATAGA |  |  |
| PINK1 | F | GCACAATGAGCCAGGAGCTA | 298 | XM 014737247.1 |
|  | R | GGGGTATTCACGCGAAGGTA |  |  |
| PARKIN | F | TCCCAGTGGAGGTCGATTCT | 218 | XM 014858374.1 |
|  | R | CCCTCCAGGTGTGTTCGTTT |  |  |
| IL-6 | F | GAGGATACCACTCCCAACAGACC | 141 | NM_001314054.1 |
|  | R | AAGTGCATCATCGTTGTTCATAA |  |  |
| IL-8 | F | CTGGCTGTGGCTCTCTTG | 132 | NM_001083951.2 |
|  | R | CAGTTTGGGATTGAAAGGTTTG |  |  |
| IL-10 | F | TGTTGTTGAACGGGTCCCTG | 757 | XM_014730883.1 |
|  | R | ACTCTTCACCTGCTCCACTG |  |  |
| IL-13 | F | AGCAGTCATTGCTCTCGCTT | 144 | XM_023616897.1 |
|  | R | CTCCACACCATGCTGCCATT |  |  |
| IL-1β | F | TATGTGTGTGATGCAGCTGTGC | 189 | NM_001082526.1 |
|  | R | GGCCACAGGTATCTTGTCAGT |  |  |
| TGF-β1 | F | ATTCCTGGCGCTACCTCAGT | 197 | NM_001081849.1 |
|  | R | GCTGGAACTGAACCCGTTGAT |  |  |
| TNF-α | F | AAGTGACAAGCCTGTAGCCC | 254 | XM_014831605.1 |
|  | R | GGTTGACCTTGGACGGGTAG |  |  |
| CHOP | F | AGCCAAAATCAGAGCCGGAA | 272 | XM_001488999.4 |
|  | R | GGGGTCAAGAGTGGTGAAGG |  |  |
| PERK | F | GTGACTGCAATGGACCAGGA | 283 | XM_023618757.1 |
|  | R | TCACGTGCTCACGAGGATATT |  |  |
| eIF2a | F | AGTCTTCAGGCATTGGCTCC | 489 | XM_001488848.6 |
|  | R | CCGAGTGGGACATGTATCGG |  |  |
| BiP | F | CTGTAGCGTATGGTGCTGCT | 122 | XM_023628864.1 |
|  | R | CATGACACCTCCCACGGTTT |  |  |
| IRE1 | F | GAATCAGACGAGCACCCGAA | 300 | XM_023652216.1 |
|  | R | TTTCTTGCAGAGGCCGAAGT |  |  |
| ATF6 | F | CAGGGTGCACTAGAACAGGG | 164 | XM_023640315.1 |
|  | R | AATGTGTCTCCCCTTCTGCG |  |  |
| XBP1 | F | TTACGCGAGAAAACTCATGGCC | 281 (unspliced)  255 (spliced) | XM_014742035.2 |
|  | R | GGGTCCAAGTTGAACAGAATGC |  |  |
| GAPDH | F | GATGCCCCAATGTTTGTGA | 250 | NM 001163856.1 |
|  | R | AAGCAGGGATGATGTTCTGG |  |  |

Sequence, product size and accession numbers of the primers set. TET2: Tet methylcytosine dioxygenase 2; TET 3: Tet methylcytosine dioxygenase 3; CXCR4: C-X-C chemokine receptor type 4; p53: tumor suppressor p53; p21: Cyclin‐Dependent Kinase Inhibitor 1A; BAX: BCl-2 associated X protein; BCl-2*:* B-cell lymphoma 2; MFN1: mitofusin 1; FIS1: mitochondrial fission 1 molecule; PINK1: PTEN-induced putative kinase; PARKIN: parkin RBR E3 ubiquitin protein ligase (PARK2); IL-6: interleukin 6; TGF-β1: transforming growth factor β1; TNF-α: tumor necrosis factor alpha; IRS: insulin receptor substrate; SREBP-1C: sterol regulatory element-binding transcription factor 1; SIRT1: sirtuin 1; GLUT-4: glucose transporter 4; FOXO1: forkhead box protein O1; CHOP: CCAAT-enhancer-binding protein homologous protein*; PERK:* protein kinase RNA-like endoplasmic reticulum kinase; eIF2a: eukaryotic translation initiation factor 2α; BiP: binding immunoglobulin protein; IRE1: inositol-requiring enzyme 1; ATF6: activating transcription factor 6; XBP1: X-box binding protein 1; GADPH: glyceraldehyde‐3‐phosphate dehydrogenase
